# The downregulation of type I IFN signaling in G-MDSCs under tumor conditions promotes their development towards an immunosuppressive phenotype

**DOI:** 10.1038/s41419-021-04487-w

**Published:** 2022-01-10

**Authors:** Yingying Sun, Xiaoqing Han, Chao Shang, Yawei Wang, Boya Xu, Shu Jiang, Yan Mo, Dake Wang, Yueshuang Ke, Xianlu Zeng

**Affiliations:** 1 The Key Laboratory of Molecular Epigenetics of Ministry of Education, Institute of Genetics and Cytology, School of Life Science, Northeast Normal University, Changchun, Jilin China; 2 Laboratory of Chemical Biology, Changchun Institute of Applied Chemistry, Chinese Academy of Sciences, Changchun, Jilin China

**Keywords:** Cancer, Immune evasion

## Abstract

Tumors modify myeloid cell differentiation and induce an immunosuppressive microenvironment. Granulocytic myeloid-derived suppressor cells (G-MDSCs), the main subgroup of myeloid-derived suppressor cells (MDSCs), are immature myeloid cells (IMCs) with immunosuppressive activity and exist in tumor-bearing hosts. The reason why these cells diverge from a normal differentiation pathway and are shaped into immunosuppressive cells remains unclear. Here, we reported that the increase of granulocyte colony-stimulating factor (G-CSF) in mouse serum with tumor progression encouraged G-MDSCs to obtain immunosuppressive traits in peripheral blood through the PI3K-Akt/mTOR pathway. Importantly, we found that downregulation of type I interferon (IFN-I) signaling in G-MDSCs was a prerequisite for their immunosuppressive effects. Suppressor of cytokine signaling (SOCS1), the action of which is dependent on IFN-I signaling, inhibited the activation of the PI3K-Akt/mTOR pathway by directly interacting with Akt, indicating that the differentiation of immunosuppressive G-MDSCs involves a transition from immune activation to immune tolerance. Our study suggests that increasing IFN-I signaling in G-MDSCs may be a strategy for reprograming immunosuppressive myelopoiesis and slowing tumor progression.

## Introduction

Avoiding immune destruction is an important characteristic of cancer cells [1]. Tumor conditions can induce the generation of multiple immunosuppressive cells (regulatory T cells (Tregs), tumor-associated macrophages, myeloid-derived suppressor cells (MDSCs), etc.) [2]. MDSCs have a phenotype like that of immature myeloid cells (IMCs). Under physiological conditions, IMCs differentiate into dendritic cells, macrophages, and granulocytes. However, MDSCs can abnormally accumulate under chronic inflammatory conditions, cancer, or autoimmunity [3]. MDSCs have been shown to inhibit antitumor immunity [4]. Upregulation of immunosuppressive molecules (such as Arg1, iNOS, and TGF-β) in MDSCs leads to suppression of T-cell proliferation and function [5]. MDSCs can also secrete some cytokines to promote tumor cells survival, proliferation and metastasis [6, 7].

In cancer, MDSC expansion and differentiation are associated with cytokines secreted by tumor cells and tumor stroma cells [8–10]. Proinflammatory mediators, such as IL-1β, IL-6, and PGE2, affect the normal differentiation of IMCs and regulate MDSC accumulation [11]. MDSCs consist of two populations: monocytic MDSCs (mo-MDSCs as CD11b+Ly6ChiLy6G–) and granulocytic MDSCs (G-MDSCs as CD11b+Ly6CloLy6G+) in mice [6]. The proportions of the two MDSC subtypes are different, and ~70–80% are G-MDSCs [12–15]. One more important point is that G-MDSCs are generally much more prevalent than mo-MDSCs in cancer [16].

Type I interferon (IFN-I) is the central antiviral cytokine that transmits signals through the widely expressed IFNAR receptor (consisting of two subunits, IFNAR1 and IFNAR2) [17]. IFN-I can induce the expression of genes called interferon-stimulated genes (ISGs). ISGs exert antiviral effects via their immunomodulatory properties or by directly interfering with viral replication [18]. Moreover, IFN-I promotes immune maturation and differentiation from an innate to an adaptive immune response [19]. Recent work showed that immunosuppressive activity of MDSCs in cancer required inactivation of IFN-I pathway [20]. Although it has been reported that downregulation of IFN-I signaling is closely related to tumor development [21–24], little is known about the mechanisms between IFN-I signaling in MDSCs and their immunosuppressive activity.

Although the function of MDSCs has been clearly studied, the development of MDSCs under tumor conditions remains to be elucidated. In fact, where and how IMCs differentiate into MDSCs are not known in detail [25, 26]. Here, we sought to clarify the process by which G-MDSCs, the main subgroup of MDSCs, obtain immunosuppressive functions and explore the mechanism of their functional transformation with tumor progression. We reported that the immunosuppressive functions of G-MDSCs were first obtained in the peripheral blood of tumor-bearing mice. Upregulated G-CSF in the serum was involved in the acquisition of immunosuppressive functions by G-MDSCs via PI3K-Akt/mTOR pathway. Most importantly, the downregulation of IFN-I signaling in G-MDSCs led to the activation of the PI3K-Akt/mTOR pathway, and SOCS1 which is dependented on IFN-I signaling regulated the PI3K-Akt/mTOR pathway via direct binding of Akt. These results indicate that the downregulation of IFN-I signaling in G-MDSCs is necessary to obtain immunosuppressive function, suggesting that upregulation of IFN-I signaling may attenuate tumor progression.

## Results

### G-MDSCs are activated in peripheral blood during tumor progression

**Fig. 1:**
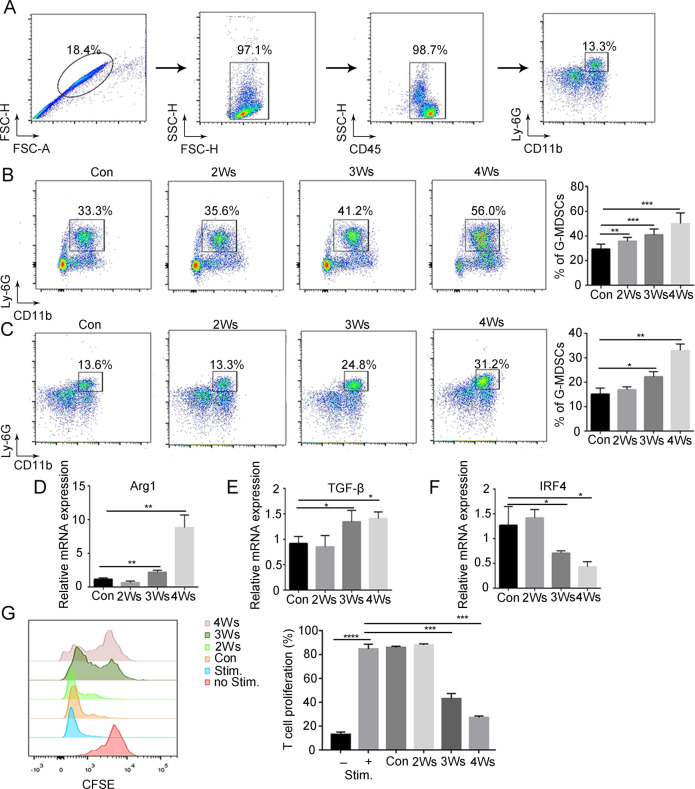
G-MDSCs are activated in peripheral blood during tumor progression. **A** Flow cytometry (FCM) showing G-MDSCs (CD45+CD11b+Ly6G+). The proportion of G-MDSCs in bone marrow (**B**) and peripheral blood (**C**) taken from mice after different durations of tumor bearing was analyzed by FCM (*n* = 6). **D**–**F** The expression levels of the Arg1, TGF-β, and IRF4 genes in G-MDSCs in peripheral blood taken from mice after different durations of tumor bearing were analyzed by Q-PCR (*n* = 4). **G** The proliferation of T cells after coculture with G-MDSCs from peripheral blood taken from mice after different durations of tumor bearing was analyzed by FCM (*n* = 4). Results are mean ± SD. Unpaired *t-*test was used to determine significance (*p < 0.05, **p < 0.01, ***p < 0.001, and ****p < 0.0001).

To address that abnormal amplification and activation of G-MDSCs under tumor conditions, B16F10 melanoma cells were injected subcutaneously into mice. The number of G-MDSCs was examined by flow cytometry (Fig. 1A). With tumor progression, G-MDSCs accumulated in bone marrow and peripheral blood (Fig. 1B, C and Supplementary Fig. 1A). To determine whether G-MDSCs have an immunosuppressive function in the early stage of development, the immunosuppressive activity of bone marrow G-MDSCs was examined. The results showed that the expression of the immunosuppressive genes Arg1 and TGFβ in bone marrow G-MDSCs did not change with tumor progression (Supplementary Fig. 1B). Moreover, bone marrow G-MDSCs showed no inhibition of CD8+ T-cell proliferation (Supplementary Fig. 1C). These results suggest that bone marrow G-MDSCs have no immunosuppressive function, which means that bona fide G-MDSCs require further activation.

Next, the immunosuppressive activity of peripheral blood G-MDSCs was examined. The expression of Arg1 and TGF-β in peripheral blood G-MDSCs began to significantly increase after 3 weeks of tumor bearing (Fig. 1D, E). IRF4 negatively regulates the immunosuppressive function of MDSCs [27] and IRF4 in peripheral blood G-MDSCs was significantly decreased after 3 weeks of tumor bearing (Fig. 1F). Then we found that peripheral blood G-MDSCs of mice bearing tumors for 3 weeks significantly inhibited T-cell proliferation (Fig. 1G), indicating that G-MDSCs acquire immunosuppressive functions in the peripheral blood. CXCR2 and CD101 can be used as markers for the maturity of polymorphonuclear cells [28]. Our results showed that the expression of CXCR2 and CD101 in peripheral blood G-MDSCs of tumor-bearing mice was reduced (Supplementary Fig. 1D, E). This result indicates that peripheral blood G-MDSCs are in an immature stage.

### Accumulation of G-MDSCs with immunosuppressive functions promotes tumor growth and metastasis

**Fig. 2:**
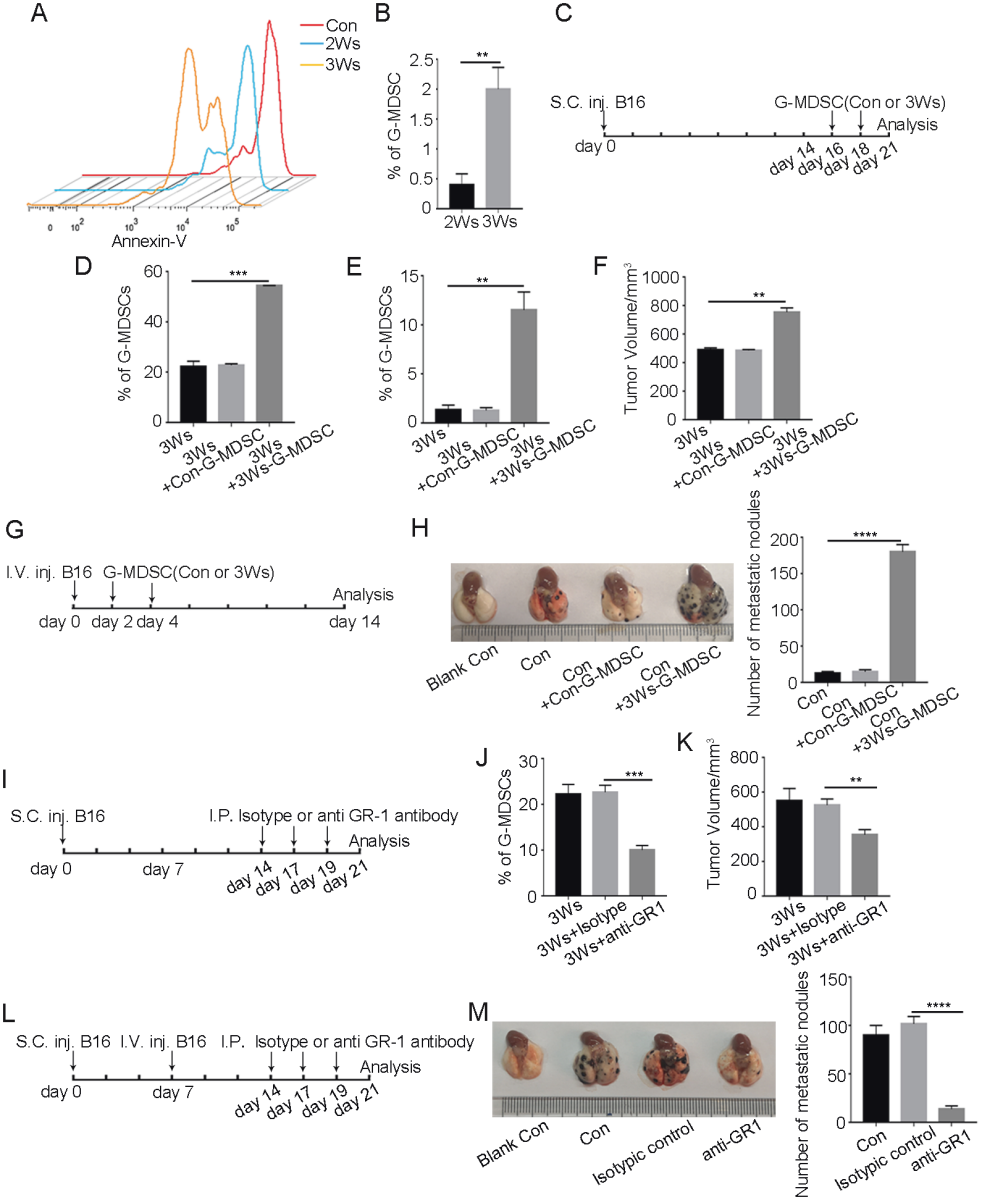
G-MDSCs with immunosuppressive functions promote tumor growth and metastasis. **A** Annexin-V expression in G-MDSCs in peripheral blood taken from mice after different durations of tumor bearing was assessed by FCM. **B** The proportion of G-MDSCs in tumor tissue taken from mice bearing tumors for 2 or 3 weeks was analyzed by FCM (*n* = 3). **C**, **G** Schematic depiction of the experimental protocol for infusion of mice with either G-MDSCs from the peripheral blood of normal mice (Con-G-MDSCs) or G-MDSCs from mice bearing tumors for 3 weeks (3Ws-G-MDSCs). Tumor-bearing mice were infused with either Con-G-MDSCs or 3Ws-G-MDSCs at the indicated time, and the proportions of G-MDSCs in peripheral blood (**D**) and tumor tissue (**E**) were analyzed by FCM (*n* = 4). **F** Tumor volume was measured after the tumor-bearing mice received either Con-G-MDSCs or 3Ws-G-MDSCs at the indicated time (*n* = 4). **H** The number of metastatic foci was detected after intravenous injection of B16F10 cells and infused with either Con-G-MDSCs or 3Ws-G-MDSCs (*n* = 4). **I**, **L** Schematic depiction of the experimental design. B16F10 cells were injected into mice through the tail vein, and the mice were treated with an anti-GR1 antibody (200 μg/I.P.) every 3 days. On day 21, lung metastasis was analyzed. **J** The proportion of G-MDSCs in peripheral blood was detected after anti-GR1 antibody treatment (*n* = 3). **K** Tumor volume was measured after anti-GR1 antibody treatment (*n* = 4). **M** The number of metastatic foci was detected after anti-GR1 antibody treatment (*n* = 4). Results are mean ± SD. Unpaired *t-*test was used to determine significance (**p < 0.01, ***p < 0.001 and ****p < 0.0001).

Polymorphonuclear cells are considered to be short-lived cells [29]. We found that the apoptosis of peripheral blood G-MDSCs was decreased under tumor conditions (Fig. 2A), thus contributing to G-MDSC activity. The tumor tissue is the final location in which G-MDSCs exert immunosuppressive functions [30]. We confirmed that G-MDSCs could be recruited into tumor tissue (Fig. 2B). To detect the effect of G-MDSCs on tumor growth and metastasis, peripheral blood G-MDSCs of normal mice or mice bearing tumors for 3 weeks were injected into mice by tail vein (Fig. 2C). In the group of mice injected with peripheral blood G-MDSCs of mice bearing tumors for 3 weeks, the number of G-MDSCs in peripheral blood and tumor tissue was significantly higher than that in other groups (Fig. 2D, E and Supplementary Fig. 2A), and a significant increase in tumor growth was observed (Fig. 2F). G-MDSCs with immunosuppressive functions also promoted tumor cells metastasis to the lung (Fig. 2G, H). After G-MDSCs were depleted by anti-GR1 antibody (Fig. 2I, J, L and Supplementary Fig. 2B), tumor growth and metastasis were inhibited (Fig. 2K, M). Overall, these results indicate that immunosuppressive G-MDSCs promote tumor growth and metastasis.

### Activation of the PI3K-Akt/mTOR pathway is related to the expression of immunosuppressive gene

**Fig. 3:**
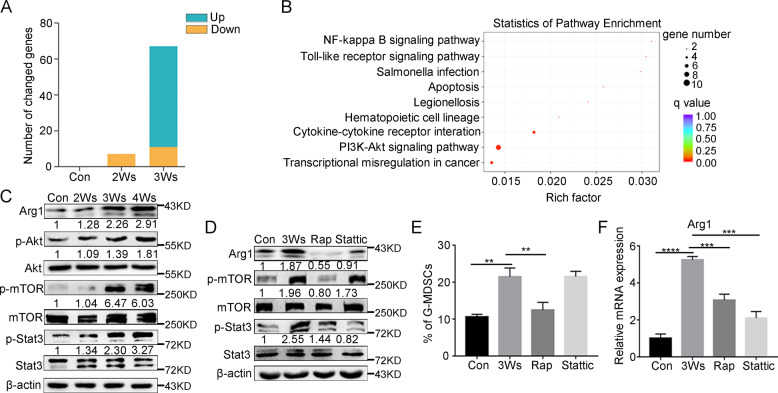
Activation of the PI3K-Akt/mTOR pathway in G-MDSCs is related to the expression of immunosuppressive gene. **A** Number of genes that changed in G-MDSCs in peripheral blood taken from tumor-bearing mice compared to normal mice at different time points. **B** Kyoto Encyclopedia of Genes and Genomes (KEGG) pathway analysis of G-MDSCs in peripheral blood taken from mice bearing tumors for different durations based on differentially expressed genes. **C** The expression of Akt, p-Akt, mTOR, p-mTOR, Stat3, and p-Stat3 in G-MDSCs in peripheral blood taken from mice bearing tumors for different durations was analyzed by western blot. **D** The expression of mTOR, p-mTOR, Stat3, and p-Stat3 in G-MDSCs in peripheral blood taken from mice bearing tumors for 3 weeks was detected by western blot after injection of rapamycin (5 mg/kg) and stattic (4 mg/kg) into the tumor-bearing mice. **E** The proportion of G-MDSCs in peripheral blood was analyzed by FCM. The mice were treated as described in **D** (*n* = 3). **F** The expression of Arg1 was detected by Q-PCR. The mice were treated as described in **D** (*n* = 3). Results are mean ± SD. Unpaired *t-*test was used to determine significance (**p < 0.01, ***p < 0.001, and ****p < 0.0001).

To explore the nature of IMC differentiation into immunosuppressive G-MDSCs, transcriptome sequencing was performed. The purity of separated G-MDSCs was detected (Supplementary Fig. 3A). Differential expression analysis of the sequencing data revealed the gene expression patterns of peripheral blood G-MDSCs from tumor-bearing mice at different stages. With tumor progression, the number of genes that changed in G-MDSCs gradually increased (Fig. 3A). Kyoto Encyclopedia of Genes and Genomes pathway analysis performed on RNA-seq data revealed an enrichment of the PI3K-Akt pathway in immunosppressive G-MDSCs (Fig. 3B). PI3K-Akt signaling regulates metabolism, growth and survival through mammalian target of rapamycin (mTOR), and it has also been recognized that the PI3K-Akt/mTOR pathway has broad roles in immune cells [31, 32]. Indeed, we observed that the phosphorylation of Akt and mTOR was upregulated in immunosppressive G-MDSCs (Fig. 3C). mTOR can further activate Stat3, which plays an important role in immunosuppression [33–35], suggesting that the PI3K-Akt/mTOR pathway is likely a driver of G-MDSC function. We next examined the effect of inhibitors (an mTOR inhibitor, rapamycin, and a Stat3 inhibitor, stattic) on G-MDSCs in vivo, and the results showed that the percentage of G-MDSCs in peripheral blood and the expression of Arg1 both decreased after injecting rapamycin (Fig. 3D–F and Supplementary Fig. 3B). Taken together, these results indicate that the PI3K-Akt/mTOR pathway promotes G-MDSCs to obtain immunosuppressive function.

### IFN-I signaling is inhibited as G-MDSCs differentiate toward an immunosuppressive phenotype

**Fig. 4:**
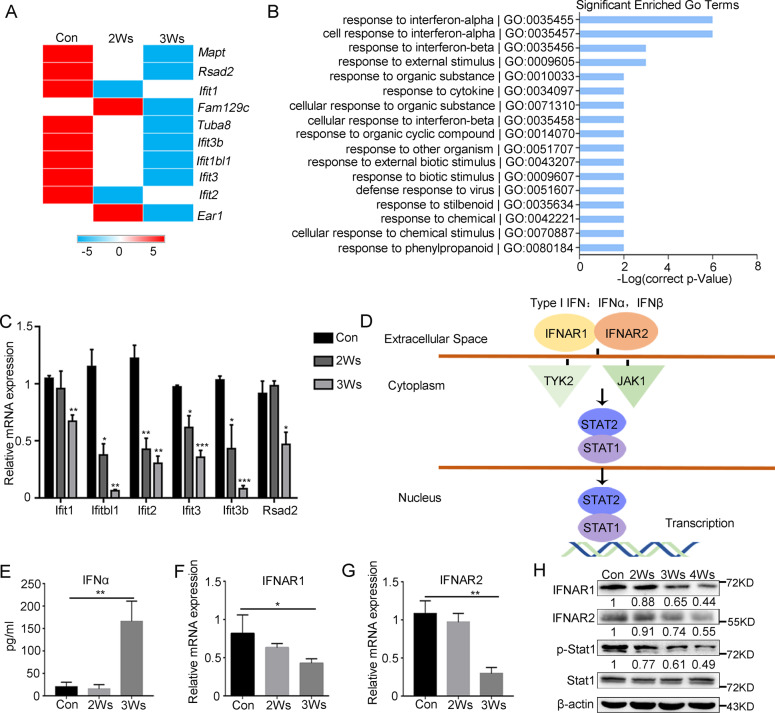
IFN-I signaling is downregulated in immunosuppressive G-MDSCs. **A** Heatmap of the downregulated genes in G-MDSCs in peripheral blood taken from mice after different durations of tumor bearing. Log10 (fold change) values were calculated from RNA-seq fragments per kilobase of transcript per million (FPKM) values. **B** Gene ontology (GO) enrichment analysis of G-MDSCs in peripheral blood taken from mice after different durations of tumor bearing based on differentially expressed genes (FC > 2). **C** Technical validation of the array data by Q-PCR (*n* = 4). **D** Schematic diagram of the IFN-I pathway. **E** The content of IFNα in serum taken from mice after different durations of tumor bearing was analyzed by ELISA (*n* = 4). **F**, **G** The expression of IFNAR1 and IFNAR2 in G-MDSCs in peripheral blood taken from mice after different durations of tumor bearing was analyzed by Q-PCR (*n* = 4). **H** The expression of IFNAR1, IFNAR2, Stat1 and p-Stat1 in G-MDSCs in peripheral blood taken from mice after different durations of tumor bearing was analyzed by western blot. Results are mean ± SD. Unpaired *t-*test was used to determine significance (*p < 0.05, **p < 0.01 and ***p < 0.001).

IFN-I has a pivotal role in promoting antitumor responses, and IFN-I signaling is essential for antiviral and immunoregulatory actions via induction of ISGs [36, 37]. The transcriptome sequencing results showed that ISGs were significantly downregulated during G-MDSCs differentiation. Gene Ontology enrichment analysis demonstrated that the differentially expressed genes (DEGs) were mainly related to the IFN-I response (Fig. 4A, B). The results were validated by Q-PCR (Fig. 4C). Downregulation of ISGs could be mapped at multiple levels in the IFN-JAK-STAT1 signal cascade (Fig. 4D). The content of IFNα was detected by ELISA. Strikingly, we observed an obvious upregulation of IFNα in serum from mice bearing tumors for 3 weeks (Fig. 4E). The loss of IFNAR could also result in the downregulation of ISGs. Indeed, IFNAR was significantly downregulated in immunosuppressive G-MDSCs (Fig. 4F, G). And the protein level of IFNAR and the Stat1 phosphorylation in G-MDSCs also decreased (Fig. 4H).

The relationship between the downregulation of IFN-I signaling and the immunosuppressive function of G-MDSCs was next investigated. The expression of Arg1 in G-MDSCs from different tissues was detected, and the results showed that Arg1 expression was gradually upregulated in bone marrow, peripheral blood and tumor tissue (Supplementary Fig. 4A). We then examined IFN-I signaling expression in G-MDSCs from different tissues, and the results showed that G-MDSCs from tumor tissue had the strongest immunosuppressive activity and the lowest IFNAR (Supplementary Fig. 4B, C) and ISGs expression (Supplementary Fig. 4D–G). In vitro stimulation with IFNα showed that the G-MDSCs from tumor tissue barely responded to IFNα (Supplementary Fig. 4H–M). These results indicate that the IFN-I signaling of immunosuppressive G-MDSCs is inhibited.

### G-CSF is required for the activation of PI3K-Akt/mTOR pathway and G-MDSC immunosuppressive function

**Fig. 5:**
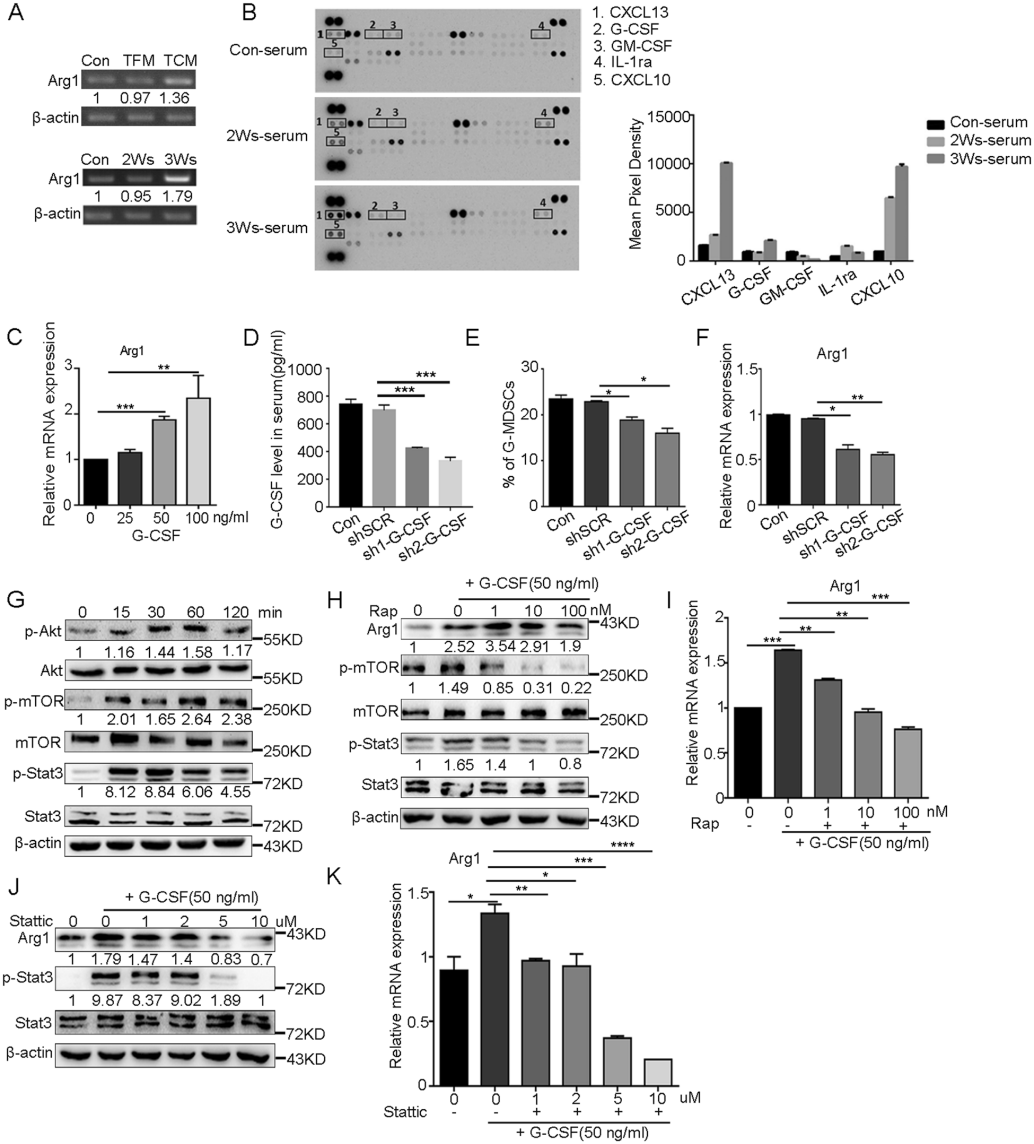
G-CSF promotes the expression of immunosuppressive genes in G-MDSCs by activating the PI3K-Akt/mTOR signaling pathway. **A** G-MDSCs taken from bone marrow of normal mice were cultured with tumor-free media (TFM), tumor cell-conditioned media (TCM) or serum from mice bearing tumors for different durations for 2 h, and the expression of Arg1 was analyzed by semi-quantitative PCR. **B** The expression profiles of various cytokines in the serum of mice bearing tumors for 0, 2, or 3 weeks were assessed using a cytokine array. **C** The expression of Arg1 in G-MDSCs taken from bone marrow of normal mice was analyzed by Q-PCR after incubation with different concentrations of G-CSF for 2 h (*n* = 5). **D** The level of G-CSF in serum was analyzed by ELISA after mice were infected with lentiviruses carrying shScramble (shSCR), sh1-G-CSF, or sh2-G-CSF (*n* = 4). **E** The proportion of G-MDSCs in peripheral blood was analyzed by FCM after mice were infected with lentiviruses carrying shSCR, sh1-G-CSF, or sh2-G-CSF (*n* = 4). **F** The expression of Arg1 in G-MDSCs from peripheral blood was analyzed by Q-PCR after mice were infected with lentiviruses carrying shSCR, sh1-G-CSF- or sh2-G-CSF (*n* = 4). **G** The expression of Akt, p-Akt, mTOR, p-mTOR, Stat3, and p-Stat3 in G-MDSCs taken from the bone marrow of normal mice was analyzed by western blot after G-CSF (50 ng/ml) was added to the culture and incubated for the indicated minutes. **H** G-MDSCs taken from the bone marrow of normal mice were cultured in vitro with G-CSF (50 ng/ml) and different concentrations of rapamycin for 2 h, and the expression of Akt, p-Akt, mTOR, p-mTOR, Stat3, and p-Stat3 was detected by western blot. **I** The expression of Arg1 was detected by Q-PCR. The cells were treated as described in **H** (*n* = 4). **J** G-MDSCs taken from the bone marrow of normal mice were cultured in vitro with G-CSF (50 ng/ml) and different concentrations of stattic for 2 h, and the expression of Stat3 and p-Stat3 was detected by western blot. **K** The cells were treated as described in **J**, and the expression of Arg1 was detected by Q-PCR (*n* = 4). Results are mean ± SD. Unpaired *t-*test was used to determine significance (*p < 0.05, **p < 0.01, ***p < 0.001 and ****p < 0.0001).

To determine the relationship between G-MDSC immunosuppressive function and tumor progression, we used conditioned medium from nontumor cells (mouse embryonic fibroblasts (MEFs)) and B16F10 and serum from tumor-bearing mice to induce G-MDSC differentiation. The results showed that the Arg1 expression in bone marrow G-MDSCs induced by tumor conditioned medium and serum from tumor-bearing mice was significantly upregulated (Fig. 5A). To identify the factors associated with this process, we analyzed the serum of mice bearing tumors for 0, 2 or 3 weeks by using a proteome profiling array. CXCL10, CXCL13, and G-CSF were present at higher concentrations in the serum of tumor-bearing mice (Fig. 5B). The source of the changed factors was detected, and the results showed that B16F10 expressed CXCL10 and CXCL13, but G-CSF was secreted by other stromal cells (Supplementary Fig. 5A). G-MDSCs expressed all the receptors of CXCL10, CXCL13, and G-CSF (CXCR3, CXCR5, and G-CSFR, respectively) (Supplementary Fig. 5B). Then, the effect of altered cytokines on the immunosuppressive function of G-MDSCs was determined in vitro. The results showed that only G-CSF increased the expression of Arg1 (Fig. 5C), and CXCL10 and CXCL13 could not directly affect the expression of Arg1 (Supplementary Fig. 5C, D). We confirmed the upregulation of G-CSF in serum of tumor mice by ELISA (Supplementary Fig. 5E). Then we generated two independent short hairpin RNAs (shRNAs) to stably decrease G-CSF levels (Fig. 5D and Supplementary Fig. 5F). After shRNA transfection in vivo, we observed a decrease in the number of G-MDSCs and a decrease in the expression of Arg1 (Fig. 5E, F and Supplementary Fig. 5G).

Next, the effect of G-CSF on the PI3K-Akt/mTOR pathway was examined. After treatment of bone marrow G-MDSCs with G-CSF in vitro, PI3K-Akt/mTOR pathway-related proteins were activated (Fig. 5G). The effect of rapamycin on G-CSF-stimulated G-MDSCs was next investigated, and the results showed that after treatment with rapamycin, Stat3 phosphorylation decreased and Arg1 expression was decreased (Fig. 5H, I). Consistent with previous reports [34, 38, 39], stattic treatment reduced the expression of Arg1 (Fig. 5J, K). These results suggest that G-CSF promotes G-MDSCs immunosuppressive functions through PI3K-Akt/mTOR pathway.

### Downregulation of IFN-I signaling is a prerequisite for G-MDSCs to obtain immunosuppressive function

**Fig. 6:**
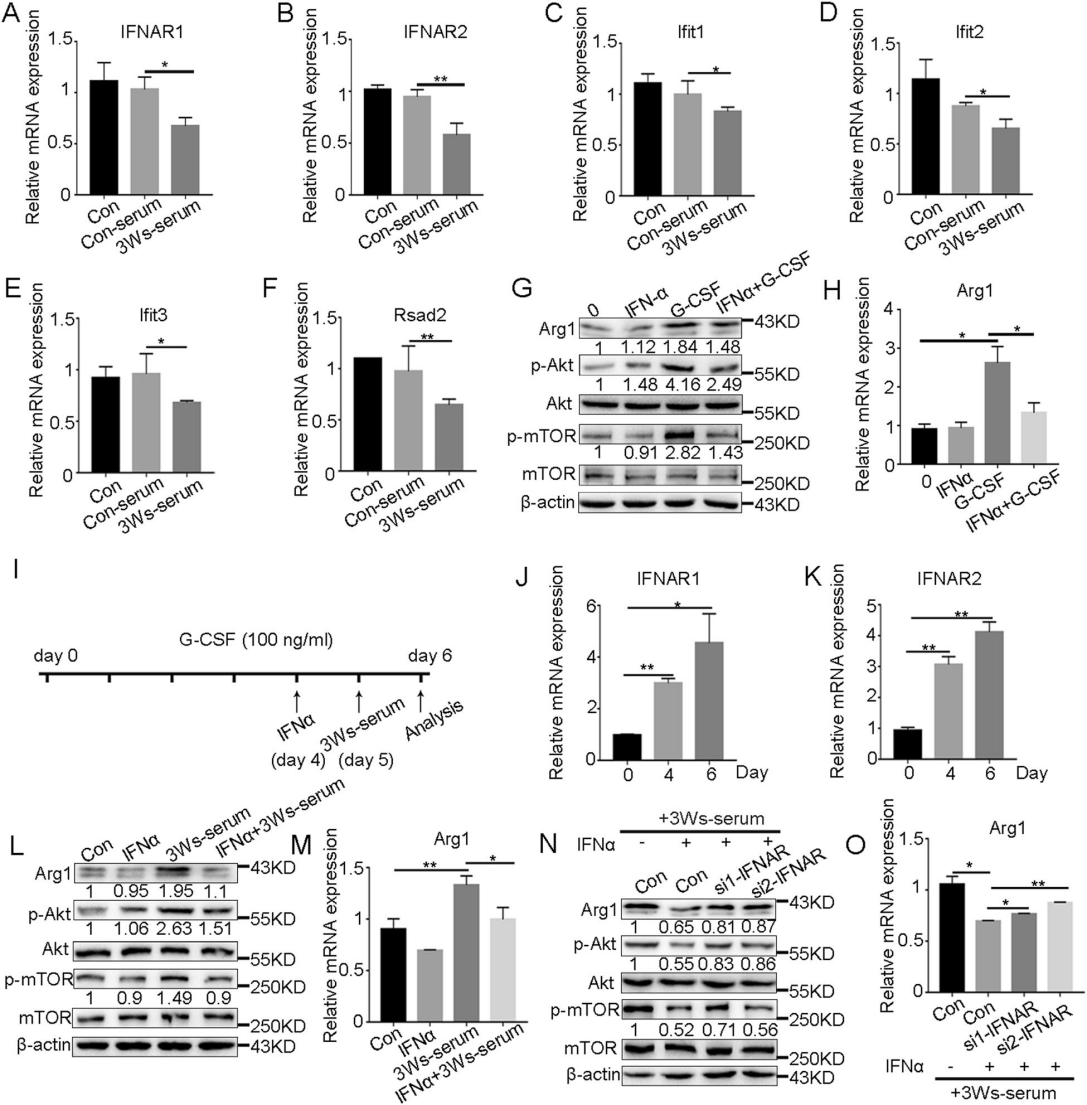
Downregulation of IFN-I signaling is a prerequisite for G-MDSCs to obtain immunosuppressive functions. **A**–**F** The expression of IFNAR and ISGs in G-MDSCs taken from the bone marrow of normal mice was analyzed by Q-PCR after incubation with serum from normal mice or the mice tumor bearing for 3 weeks (*n* = 4). **G** The expression of Arg1, Akt, p-Akt, mTOR, and p-mTOR in G-MDSCs taken from the bone marrow of normal mice was analyzed by western blot after incubation with IFNα (100 ng/ml) or G-CSF (50 ng/ml) for 2 h or stimulation with IFNα for 1 h and subsequent incubation with G-CSF for 2 h (IFNα + G-CSF). **H** The expression of Arg1 in G-MDSCs taken from the bone marrow of normal mice was analyzed by Q-PCR. The cells were treated as described in **G** (*n* = 4). **I** Schematic depiction of the experimental design. 32D clone 3 cell maturation was induced with G-CSF. IFNα treatment was performed on day 4, and 3Ws-serum was added on day 5. **J**, **K** The expression of IFNAR in 32D clone 3 cells after incubation with G-CSF was analyzed by Q-PCR (*n* = 3). **L** The expression of Arg1, Akt, p-Akt, mTOR, and p-mTOR in 32D clone 3 cells was analyzed by western blot after incubation with IFNα (100 ng/ml) or 3Ws-serum for 48 h or stimulation with IFNα for 24 h and subsequent incubation with 3W serum for 24 h (IFNα + 3Ws-serum). **M** The expression of Arg1 in 32D clone 3 cells was analyzed by Q-PCR. The cells were treated as described in **L** (*n* = 4). **N** The expression of Arg1, Akt, p-Akt, mTOR, and p-mTOR in 32D clone 3 cells was analyzed by western blot after incubation with IFNα (100 ng/ml) for 24 h and subsequent incubation with 3Ws-serum for 24 h or after IFNAR interference followed by stimulation with IFNα for 24 h and subsequent incubation with 3Ws-serum for 24 h (si-IFNAR + IFNα). **O** The expression of Arg1 in 32D clone 3 cells was analyzed by Q-PCR The cells were treated as described in **N** (*n* = 4). Results are mean ± SD. Unpaired *t-*test was used to determine significance (*p < 0.05 and **p < 0.01).

We then explored whether G-CSF affects IFN-I signaling in G-MDSCs. The expression of IFNAR and ISGs in bone marrow G-MDSCs was detected after stimulation with G-CSF in vitro. The results showed that G-CSF could not downregulate the expression of IFNAR (Supplementary Fig. 6A, B) and ISGs (Supplementary Fig. 6C–F). When stimulated with the serum of tumor-bearing mice, G-MDSCs showed significantly downregulated expression of IFNAR (Fig. 6A, B) and ISGs (Fig. 6C–F). Because the ISGs expression in G-MDSCs began to decrease after 2 weeks of tumor bearing, we speculated that downregulation of IFN-I signaling may be required for G-MDSCs to obtain immunosuppressive functions. We first stimulated bone marrow G-MDSCs with IFNα and then stimulated them with G-CSF. The results showed that when G-MDSCs were first stimulated by IFNα, G-CSF did not activate the PI3K-Akt/mTOR pathway (Fig. 6G). Similarly, the Arg1 expression was inhibited (Fig. 6H). 32D clone 3 is a mouse progenitor cell line that can be induced to differentiate into neutrophils after the addition of stem cell factor and IL-3 and the subsequent addition of G-CSF in vitro [40, 41]. We evaluated the effects of IFN-I signaling on tumor-induced G-MDSC differentiation, and the scheme of the experimental design is shown in Fig. 6I. With the differentiation of 32D cells into neutrophils, IFNAR was upregulated (Fig. 6J, K), and the upregulation of IFN-I signaling (Supplementary Fig. 7A–D) was followed by the downregulation of the PI3K-Akt/mTOR pathway and Arg1 expression (Fig. 6L, M). Interference with IFNAR (Supplementary Fig. 7E, F) attenuated IFN-I signaling (Supplementary Fig. 7G–J) and thus promoted the upregulation of the PI3K-Akt/mTOR pathway and Arg1 expression (Fig. 6N, O). The above results indicate that the downregulation of IFN-I signaling in G-MDSCs under tumor conditions promotes their development toward an immunosuppressive phenotype. We also detected the expression of IFN-I signaling in mo-MDSCs, and the results showed that the IFN-I signaling in mo-MDSCs was also downregulated (Supplementary Fig. 8), suggesting that the downregulation of IFN-I signaling under tumor conditions is necessary for the formation of an immunosuppressive microenvironment.

### SOCS1, which is dependent on IFN-I signaling, regulates the activation of the PI3K-Akt/mTOR pathway

**Fig. 7:**
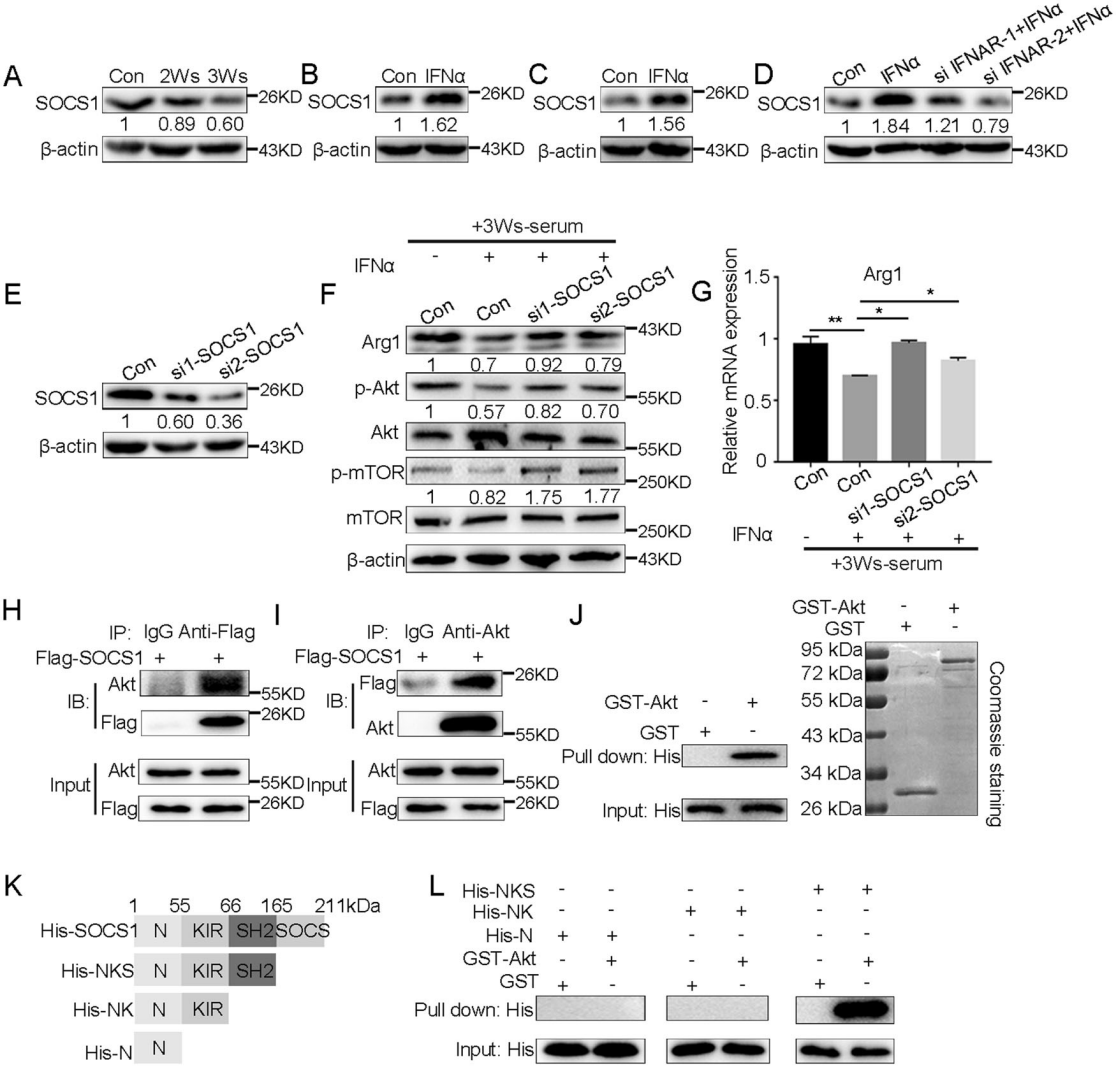
IFN-I-dependent SOCS1 can regulate the activation of PI3K-Akt/mTOR pathway. **A** The expression of SOCS1 in G-MDSCs in peripheral blood taken from mice after different durations of tumor bearing was analyzed by western blot (*n* = 5). The expression of SOCS1 in G-MDSCs from the bone marrow of normal mice (**B**) and 32D clone 3 cells (**C**) was analyzed by western blot after incubation with IFNα for 24 h (*n* = 5). **D** The expression of SOCS1 in 32D clone 3 cells stimulated with IFNα was analyzed by western blot after IFNAR interference (*n* = 4). **E** 32D clone 3 cells were transfected with siRNA targeting SOCS1 or control for 36 h, and the interference efficiency was tested (*n* = 4). **F** The expression of Arg1, Akt, p-Akt, mTOR, and p-mTOR in 32D clone 3 cells was analyzed by western blot after incubation with IFNα (100 ng/ml) for 24 h and subsequent incubation with 3W serum for 24 h or after SOCS1 interference followed by stimulation with IFNα for 24 h and subsequent incubation with 3Ws-serum for 24 h (si-SOCS1 + IFNα). **G** The expression of Arg1 in 32D clone 3 cells was analyzed by Q-PCR. The cells were treated as described in **F** (*n* = 4). **H**, **I** Co-IP assays were performed to assess the interaction between SOCS1 and Akt after 32D clone 3 cells were transfected with Flag-SOCS1 for 30 h. **J** A GST pull-down assay of Akt was conducted using the indicated proteins expressed in bacteria. His-tagged SOCS1 was expressed in bacteria, and the lysates were incubated with purified GST or GST-fused Akt. Bound proteins were separated by SDS-PAGE and immunoblotted with an anti-His antibody. **K** Schematics of the His-tagged SOCS1 expression plasmid and its domains. **L** A GST pull-down assay of Akt was conducted using the indicated proteins expressed in bacteria. Different domains of SOCS1 with a His tag were expressed in bacteria, and the lysates were incubated with purified GST or GST-fused Akt. The levels of pulled down His were detected by western blot. Results are mean ± SD. Unpaired *t-*test was used to determine significance (*p < 0.05, **p < 0.01, ***p < 0.001 and ****p < 0.0001).

Based on the above findings, we explored the mechanism by which IFN-I signaling regulates PI3K-Akt pathway in G-MDSCs. Suppressor of cytokine signaling (SOCS) members are thought to act as classic negative feedback inhibitors, which are induced by cytokines, and subsequently inhibit the function of cytokines [42, 43]. SOCS1 can not only regulate IFN-I signaling via negative feedback [17] but also inhibit the PI3K-Akt pathway [44, 45]. Our results showed that with tumor progression, the expression of SOCS1 in peripheral blood G-MDSCs was gradually downregulated (Fig. 7A and Supplementary Fig. 9A). We used IFNα to stimulate bone marrow G-MDSCs and 32D cells in vitro to detect the expression of SOCS1, and the results showed that IFNα could significantly induce the expression of SOCS1 (Fig. 7B, C and Supplementary Fig. 9B, C). Consistently, after interference with IFNAR, the expression of SOCS1 was significantly downregulated (Fig. 7D and Supplementary Fig. 9D). We speculated that the expression of SOCS1 induced by IFN-I is related to the PI3K-Akt pathway in G-MDSCs. To assess this possibility, we designed siRNA to interfere with SOCS1 in 32D cells and detected the interference efficiency (Fig. 7E and Supplementary Fig. 9E). Then, the effects of SOCS1 on the PI3K-Akt/mTOR pathway activation and Arg1 expression were examined. The results showed that the phosphorylation of Akt and mTOR and the expression of Arg1 in 32D cells were upregulated after interference with SOCS1 (Fig. 7F, G). To explore how SOCS1 inhibits the activation of PI3K-Akt pathway, a co-immunoprecipitation (co-IP) assay was performed. The results showed that SOCS1 and Akt existed in the same complex (Fig. 7H, I). To confirm whether SOCS1 directly binds to Akt, we prepared His-SOCS1 and GST-Akt constructs from bacteria, and in vitro GST pull-down assays showed that SOCS1 physically associates with Akt through direct interaction (Fig. 7J). Next, we assessed which domain of SOCS1 interacts with Akt. SOCS1 consists of an N-terminal region, a kinase inhibitory region (KIR), a central SH2 region and a carboxy-terminal SOCS box [46]. We generated His-tagged deletion mutants containing the N-terminal region, N-terminal region-KIR, and N-terminal region-KIR-SH2 domains (Fig. 7K), and pull-down assays were performed. The results showed that GST-Akt could pull-down His-tagged mutants only in the presence of the SOCS1 SH2 domain (Fig. 7L), indicating that the SH2 domain of SOCS1 is required for inhibiting PI3K-Akt/mTOR signaling.

## Discussion

The main function of MDSCs is immunosuppression, but this function is not innate. It has been reported that MDSCs at the early stage of tumors do not exhibit immunosuppression [47, 48]. It is not clear where and how MDSCs obtain immunosuppressive functions. Here, we reported that G-MDSCs, the main subgroup of MDSCs, initiated immunosuppressive functions in the peripheral blood under tumor conditions. Our research deeply analyzed the specific processes by which G-MDSCs are activated and provides new evidence that MDSCs with immunosuppressive functions need to undergo two steps, amplification and activation.

Tumor and/or stromal cells secrete inflammatory mediators to influence myeloid cells differentiation [9, 10, 49]. G-CSF has multiple effects on polymorphonuclear cells, including regulation of proliferation and mobilization [50]. In many tumors, high levels of G-CSF are produced [50–52]. Our results indicate that the upregulation of G-CSF participates in the initiation of G-MDSCs immunosuppressive function. It has been reported that the absence of PI3Kγ promoted the transformation of macrophages into antitumor M1 macrophages [53], and PI3Kγ signaling promoting immune suppression in cancer [54]. The role of the PI3K-Akt/mTOR axis in G-MDSC differentiation remains largely unstudied. Here, we showed that the PI3K-Akt/mTOR signaling of peripheral blood G-MDSCs is activated and that G-CSF promotes the acquisition of immunosuppressive functions by G-MDSCs by activating the PI3K-Akt/mTOR pathway, suggesting that inhibition of PI3K-Akt/mTOR can inhibit G-MDSC immunosuppressive function and improve immunosuppressive tumor microenvironment.

The markers of neutrophils and G-MDSCs are identical. Neutrophils are considered as important effectors of the innate immune system [55]. In cancer, neutrophils undergo a phenotypic change regarded as “alternating activation” and are called tumor-associated neutrophils (TANs) [56]. Both TANs and G-MDSCs can exert immunosuppressive functions and are very similar to each other [57]. The only difference between the two may be maturity, however, some studies have pointed out that the suppressive functions of immature and mature neutrophils is a pathological response to tumorigenesis rather than a completely separate granulocytic population [28]. All told, it is necessary to study the immunosuppressive function of these cells in depth, and whether these cells are called G-MDSC or TAN is less important. IFN-I is related to the maturity of neutrophils and the development of immune function [58]. The role of IFN-I in tumor immunosuppression remains controversial. Chronic IFNα promotes MDSC accumulation [59], and IFNα can also promote the expression of PD-L1 and PD-1, which contributes to immunosuppression [60]. Conversely, stimulator of interferon genes (STING) inhibits MDSC differentiation by activating IFN-I signaling in Epstein-Barr virus-associated nasopharyngeal carcinoma [61]. Based on our findings, although the IFNα level in mouse serum increased with tumor progression, the IFN-I signaling in G-MDSCs is downregulated and its downregulation during G-MDSC differentiation was related to the acquisition of immunosuppressive function. We found that SOCS1, a classical negative feedback regulator, which depends on IFN-I signaling, can directly bind with Akt through SH2 domain to inhibit the immunosuppressive PI3K-Akt/mTOR signaling pathway in G-MDSCs. The downregulation of IFN-I signaling under tumor conditions leads to the downregulation of SOCS1, which causes the activation of the PI3K-Akt/mTOR pathway.

The IFNAR expression is essential for IFN-I signal transduction. In our study, the IFNAR expression in immunosuppressive G-MDSCs decreased, as a result, the response of G-MDSCs to IFN-I was reduced. Our results indicate that the downregulation of IFNAR promotes the activation of PI3K-Akt/mTOR, which is related to the immunosuppressive function of G-MDSCs and we further clarifies the specific mechanism by which tumor conditions induce immune activation to tilt toward immunosuppression. Previous studies have revealed that VEGF or IFN-α/β promotes the ubiquitination and degradation of IFNAR1 and ensuing restriction of cellular responses to IFN-I [62, 63]. Activation of p38 kinase is also required for IFNAR1 ubiquitination and degradation [64]. In our study, although G-CSF promotes the initiation of G-MDSCs immunosuppressive function through PI3K-Akt/mTOR pathway, only G-CSF is not enough, G-CSF can not downregulate IFN-I signaling in G-MDSCs. It remains to be elucidated what factors in the serum under tumor conditions contribute to the downregulation of IFNAR.

**Fig. 8:**
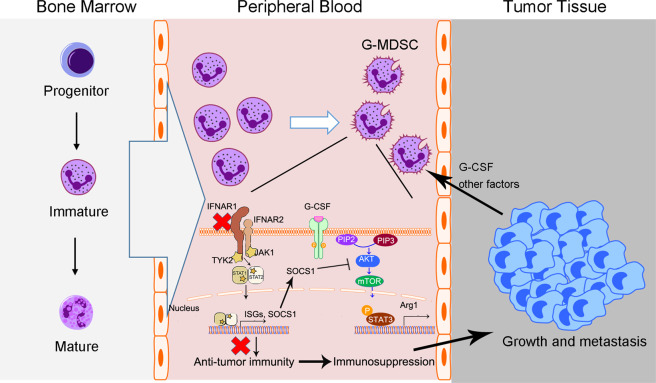
Schematic illustration of the proposed model. G-MDSCs abnormally expanded under tumor conditions obtain immunosuppressive functions in peripheral blood and ultimately promote tumor growth and metastasis. The activation of PI3K-Akt/mTOR pathway is related to the immunosuppressive function of G-MDSCs. The downregulation of IFN-I signaling in G-MDSCs leads to the activation of PI3K-Akt/mTOR pathway. SOCS1, which depends on IFN-I signaling, regulates the activation of PI3K-Akt/mTOR pathway by directly interacting with Akt.

In summary, our results show that G-MDSCs initiates immunosuppressive functions in the peripheral blood of tumor-bearing mice and that G-CSF participates in the acquisition of G-MDSC immunosuppressive functions by activating the PI3K-Akt/mTOR pathway. It is worth noting that the downregulation of IFN-I signaling in G-MDSCs is a prerequisite for their immunosuppressive function. The downregulation of IFN-I signaling in G-MDSCs leads to the activation of the PI3K-Akt/mTOR pathway by downregulating SOCS1 (Fig. 8). The results indicate that the generation of immunosuppressive G-MDSCs under tumor conditions involves a transformation from immune activation to immune tolerance, providing a mechanistic insight for combating the immunosuppressive microenvironment.

## Materials and methods

### Mice and cell lines

C57BL/6J mice (8–10 weeks old) were purchased from Beijing Vital River Laboratory Animal Technology Co., Ltd. (Beijing, China). B16F10 cells (1 × 10^6^) were implanted subcutaneously in female mice (*n* = 4–6 mice/group). Tumor growth was measured using calipers, and tumor volume was calculated as follows: *V* = (length × width^2^) × 0.5. For all drug efficacy studies, Rapamycin and Stattic treatments (five times per week) were initiated at 2 weeks of tumor-bearing. All mice were housed in specific pathogen-free conditions. All animal procedures and studies were conducted in accordance with the Institutional Animal Care and Use Committee guidelines. Mouse melanoma B16F10 cells, MEF cells, human embryonic kidney HEK-293T and 32D clone 3 cells were purchased from American Type Culture Collection. B16F10, MEF and HEK-293T cells were grown in DMEM supplemented with 10% heat-inactivated FBS (HyClone, Logan, UT, USA) and 1% penicillin/streptomycin. 32D clone 3 cells were maintained in RPMI 1640 medium supplemented with 10% heat-inactivated FBS and 10% mouse interleukin IL-3 (213–13, Peprotech) culture supplement. All cells were routinely validated for lack of Mycoplasma infection with LookOut Mycoplasma PCR Detection Kit (Sigma, Saint Louis, MO, USA) and used within 10 passages.

### Reagents and antibodies

Recombinant murine CXCL10 (250-16), CXCL13 (250-24), G-CSF (250-05), and GM-CSF (315-03) were purchased from PeproTech (Rocky Hill, NJ, USA). Directly conjugated anti-mouse mAbs CD11b-FITC (M1/70), CD45-PE/Cy7 (30-F11), Ly6G-APC/Cy7 (1A8), Ly6C-PE (AL-21) were from BD Pharmingen (San Jose, CA, USA). Carboxyfluorescein succinimidyl ester (CFSE) was from Molecular Probes (Eugene, OR, USA). The Annexin-V-APC apoptosis analysis kit (AO2001-11P-G) was obtained from Sungene (Tianjin, China). The mouse cytokine array panel A (ARY006) was purchased from R&D Systems (Minneapolis, MN, USA). The MDSC isolation kit was from Miltenyi Biotec (Bergisch Gladbach, Germany) and the streptavidin particles were from BD IMag (San Jose, CA, USA). Anti-p-Akt (Ser473, #4060), anti-Akt (#4691), anti-p-mTOR (Ser2448, #5536), anti-mTOR (#2983), anti-p-STAT3 (Tyr705, #9145) Abs were purchased from Cell Signal Technology (Danvers, MA). Anti-STAT3 (F-2) Abs was purchased from Santa Cruz Biotechnology (Santa Cruz, CA). Rapamycin (HY-10219) and Stattic (HY-13818) were purchased from Med Chem Express (New Jersey, USA).

### Flow cytometry

Single-cell suspensions from bone marrow and peripheral blood were treated with hypotonic lysis buffer to eliminate erythrocytes, and stained with fluorochrome-conjugated antibodies at 4 °C for 30 min. Following tumor tissues processing, single-cell suspensions were resuspended in FC blocked (CD16/32, BD #553141) on ice and then incubated with antibody master mix. Dead cells in tumor tissue were identified by staining with 7-AAD (Biolegend) according to the manufacturer’s instructions. For apoptosis analysis, cells were stained with surface marker followed by Annexin-V staining in binding buffer (Biolegend). The treated cells were tested by flow cytometry using a FACSCanto II (BD Biosciences, San Jose, CA, USA), and the data were analyzed with FACSDiva software (BD Biosciences) and Flow Jo 7.6.1 software.

### Cell isolation

Bone marrow cells were isolated by flushing the femurs and tibias with PBS supplemented with 2% FBS. Splenocytes were prepared by grinding the spleen against a 70-μm nylon mesh. CD8^+^ T cells were isolated from splenocytes using the Mouse CD8 T-cell Isolation Kit (Biolegend). To obtain single cell from tumors, tissues were minced and subjected to 1 h enzymatic digestion using 0.5 mg/ml collagenase IV (Sigma) and 0.01 mg/ml DNase I (Sigma) in RPMI 1640 supplemented with 2% FBS at 37 °C. Single-cell suspensions were passed through a 70-μm mesh followed by erythrocytes removal using ammonium chloride lysis buffer. For the isolation of G-MDSCs, cells were labeled with biotinylated antibody to Ly6G (Miltenyi Biotec), incubated with streptravidin-coated microbeads (Miltenyi Biotec) and separated on MACS columns **(**Miltenyi Biotec**)**. The purity and viability of the sorted cells is over 90%.

### In vitro T-cell proliferation assay

CD8+ T cells (2 × 10^5^) from normal mice were pre-stained with 1 μM CFSE at room temperature for 5 min and then quenched with 10% FBS. The treated CD8+ T cells were plated in U bottom 96-well plates in triplicates and stimulated with the antibodies of anti-CD3 (0.5 μg/ml) and anti-CD28 (1 μg/ml) in the presence of the same number of G-MDSCs. On day 3 of the coculture, cells were collected and evaluated by flow cytometry.

### RNA extraction and Q-PCR

Total RNA was extracted with TRIzol (Invitrogen), and the cDNA was synthesized with reverse transcriptase (Thermo Fisher Scientific, Waltham, MA, USA). The cDNA was then used as template for semi-quantitative or quantitative PCR analysis with Power SYBR Green PCR Master Mix (Thermo Fisher, Waltham, MA) using the StepOne Plus Real Time PCR System (Applied Biosystems, Foster City, CA). Q-PCR data were analyzed by the ΔΔCt method and relative expression of mRNA was normalized to β-actin. The primer sequences used were listed in Supplementary Table 1.

### Tumor metastasis assay in vivo

In total, 2 × 10^5^ B16F10 cells were injected via the tail vein into C57BL/6J mice and lung metastasis incidence was analyzed about 2 weeks later. To determinate the effects of G-MDSCs, C57BL/6J mice were intravenously injected with 2 × 10^5^ B16F10 cells, and followed immediately, 2 and 4 days later, by injection of 2 × 10^6^ G-MDSC or mice were treated with intraperitoneal injections of 200 μg in 100 μl of rat IgG2b isotopic control or anti-GR1 antibody.

### Cytokine array and enzyme-linked immunosorbent assay (ELISA) of serum

Mouse peripheral blood samples were centrifuged at 3000 rpm for 10 min. Serum was collected, aliquoted, and stored at −80 °C. Expression of cytokines in mouse serum was evaluated using mouse cytokine antibody array Panel A (ARY006, R&D Systems). The level of IFNα and VEGF in serum was determined using ELISA kits (eBioscience) according to manufacturer’s instructions.

### Construction of the lentiviral expression plasmid and gene transfection

Synthesized short hairpin (sh) RNAs sequences were cloned into pLVTHM vector, and the constructed plasmids or shCtrl plasmid were transfected into HEK-293T cells, together with the packaging plasmid psPAX2 and the envelope plasmid pMD2.G (both from Addgene) using Lipofectamine 2000 reagent (Invitrogen). The collected supernatant was concentrated and intrapleurally injected into 2-week tumor-bearing mice four times every other day. The relative sequences used were listed in Supplementary Table 2.

### RNA-seq analysis

For RNA sequencing (RNA-seq), peripheral blood G-MDSCs was collected from normal mice, mice bearing tumor for 2 and 3 weeks. The cellular RNA was extracted using Trizol reagent followed by a genomic DNA elimination step. RNA purity was assessed using the kaiaoK5500® Spectrophotometer (Kaiao, Beijing, China). The RNA integrity and concentration was assessed using a RNA Nano 6000 Assay Kit of the Bioanalyzer 2100 system (Agilent Technologies, CA, USA). Library construction and sequencing on an Illumina HiSeq 2500 instrument was performed at Annoroad Gene Technology Corporation (Beijing, China). Bowtie2 v2.2.3 was used to build the genome index, and clean data was then aligned to the reference genome using HISAT2 v2.1.0. The level of gene expression was quantified using a software package called FPKM (Fragments Per Kilobase Millon Mapped Reads). DEGseq v1.18.0 was used for differential gene expression analysis. Overall changes were considered significant if they passed the FDR threshold of <5%. RNA sequencing of the raw data and processed expression data for this study have been deposited in the NCBI Gene Expression Omnibus and are accessible through GEO Series accession number: GSE141652.

### Western blot

Cells were harvested and solubilized in RIPA buffer containing protease and phosphatase inhibitors. 30 μg protein was electrophoresed on Biorad precast gradient gels and electroblotted onto PVDF membranes. Proteins were detected by incubation with 1:1000 dilutions of primary antibodies, washed and incubated with Goat anti-rabbit-HRP antibodies or Goat anti-mouse-HRP antibodies and detected after incubation with a chemiluminescent substrate.

### siRNA-mediated interference

For RNA interference experiments, scramble control, or smart-pool siRNA respectively against IFNAR and SOCS1 were transfected into 32D clone 3 cells using Lipofectamine 3000 according to the manufacturer’s instructions. siRNA duplex oligoribonucleotides were synthesized by GenePharma (Shanghai, China). The sense sequences were as follows: si1-IFNAR, 5′-GUCUGAAAGUUUUGAUAGATT-3′; si2-IFNAR, 5′-CCGUUGUACUCUAGUACUUTT-3′; si1-SOCS1, 5′-CCAGUUUAGGUAAUAAACUTT-3′ and si2-SOCS1, 5′-ACACUCACUUCCGCACCUUTT-3′.

### Co-immunoprecipitation (Co-IP) assay

The cell lysates were extracted after transfection of flag-SOCS1 plasmid into 32D cells for 30 h. Cell extracts were cleared with protein G beads for 1 h at 4 °C before being incubated with 2 μg of monoclonal anti-Akt or FLAG antibodies, or, alternatively, the same amount of IgG, overnight at 4 °C. After washing, the precipitated proteins were analyzed by immunoblotting.

### GST pull-down assay

GST-Akt was cloned into the vectors pGEX-4T-2, and His-SOCS1 was cloned into pET-28a(+). *E. coli* BL21 were transformed with pGEX-4T-2-Akt and pET-28a(+)-SOCS1 vectors. GST-Akt and His-SOCS1 protein expression were induced with IPTG and purified using Glutathione Agarose 4B (Cytiva, #17075601) and Ni-NTA Agarose beads (Beyotime, #P2218) according to standard protocols. Purified GST-Akt and His-SOCS1 were incubated in reaction buffer at 4 °C for 1 h, and then with GST-beads overnight at 4 °C. Afterwards, the beads were washed four times with low-salt buffer, and bound proteins were eluted and subjected to immunoblot analysis.

### Statistics

Statistical and graphical analyses were performed using Graphpad Prism. Results are presented as means ± SD. *t*-Test was performed using GraphPad Prism version 6 software. Differences were considered to be statistically significant when *p* values were <0.05. All experiments were independently repeated at least three times.

## Supplementary information

Supplementary Figure legends

Supplementary Fig. 1

Supplementary Fig. 2

Supplementary Fig. 3

Supplementary Fig. 4

Supplementary Fig. 5

Supplementary Fig. 6

Supplementary Fig. 7

Supplementary Fig. 8

Supplementary Fig. 9

Supplementary Table 1

Supplementary Table 2

## Data Availability

All study data are included in the article and/or supporting information.

## References

[1] Hanahan D, Weinberg RA (2011). Hallmarks of cancer: the next generation. Cell..

[2] Fox BA, Sanders KL, Chen S, Bzik DJ (2013). Targeting tumors with nonreplicating Toxoplasma gondii uracil auxotroph vaccines. Trends Parasitol.

[3] Sinha P, Chornoguz O, Clements VK, Artemenko KA, Zubarev RA, Ostrand-Rosenberg S (2011). Myeloid-derived suppressor cells express the death receptor Fas and apoptose in response to T cell-expressed FasL. Blood..

[4] Gabrilovich DI, Nagaraj S (2009). Myeloid-derived suppressor cells as regulators of the immune system. Nat Rev Immunol.

[5] Ostrand-Rosenberg S, Sinha P (2009). Myeloid-derived suppressor cells: linking inflammation and cancer. J Immunol.

[6] Talmadge JE, Gabrilovich DI (2013). History of myeloid-derived suppressor cells. Nat Rev Cancer.

[7] Qu P, Wang LZ, Lin PC (2016). Expansion and functions of myeloid-derived suppressor cells in the tumor microenvironment. Cancer Lett.

[8] Tesi RJ (2019). MDSC; the most important cell you have never heard of. Trends Pharm Sci.

[9] Giallongo C, Romano A, Parrinello NL, La Cava P, Brundo MV, Bramanti V (2016). Mesenchymal stem cells (MSC) regulate activation of granulocyte-like myeloid derived suppressor cells (G-MDSC) in chronic myeloid leukemia patients. PLoS ONE.

[10] Wang J, De Veirman K, Faict S, Frassanito MA, Ribatti D, Vacca A (2016). Multiple myeloma exosomes establish a favourable bone marrow microenvironment with enhanced angiogenesis and immunosuppression. J Pathol.

[11] Groth C, Hu X, Weber R, Fleming V, Altevogt P, Utikal J (2019). Immunosuppression mediated by myeloid-derived suppressor cells (MDSCs) during tumour progression. Br J Cancer.

[12] Movahedi K, Guilliams M, Van den Bossche J, Van den Bergh R, Gysemans C, Beschin A (2008). Identification of discrete tumor-induced myeloid-derived suppressor cell subpopulations with distinct T cell-suppressive activity. Blood..

[13] Lang S, Bruderek K, Kaspar C, Hoing B, Kanaan O, Dominas N (2018). Clinical relevance and suppressive capacity of human myeloid-derived suppressor cell subsets. Clin Cancer Res.

[14] Tian X, Tian J, Tang X, Rui K, Zhang Y, Ma J (2015). Particulate beta-glucan regulates the immunosuppression of granulocytic myeloid-derived suppressor cells by inhibiting NFIA expression. Oncoimmunology..

[15] Zheng Y, Tian X, Wang T, Xia X, Cao F, Tian J (2019). Long noncoding RNA Pvt1 regulates the immunosuppression activity of granulocytic myeloid-derived suppressor cells in tumor-bearing mice. Mol Cancer.

[16] Youn JI, Nagaraj S, Collazo M, Gabrilovich DI (2008). Subsets of myeloid-derived suppressor cells in tumor-bearing mice. J Immunol.

[17] Porritt RA, Hertzog PJ (2015). Dynamic control of type I IFN signalling by an integrated network of negative regulators. Trends Immunol.

[18] Lercher A, Bhattacharya A, Popa AM, Caldera M, Schlapansky MF, Baazim H (2019). Type I interferon signaling disrupts the hepatic urea cycle and alters systemic metabolism to suppress T cell function. Immunity..

[19] Parker BS, Rautela J, Hertzog PJ (2016). Antitumour actions of interferons: implications for cancer therapy. Nat Rev Cancer.

[20] Alicea-Torres K, Sanseviero E, Gui J, Chen J, Veglia F, Yu Q (2021). Immune suppressive activity of myeloid-derived suppressor cells in cancer requires inactivation of the type I interferon pathway. Nat Commun.

[21] Snell LM, McGaha TL, Brooks DG (2017). Type I interferon in chronic virus infection and cancer. Trends Immunol.

[22] Osborn JL, Greer SF (2015). Metastatic melanoma cells evade immune detection by silencing STAT1. Int J Mol Sci.

[23] Ortiz A, Gui J, Zahedi F, Yu P, Cho C, Bhattacharya S (2019). An interferon-driven oxysterol-based defense against tumor-derived extracellular vesicles. Cancer Cell.

[24] Critchley-Thorne RJ, Simons DL, Yan N, Miyahira AK, Dirbas FM, Johnson DL (2009). Impaired interferon signaling is a common immune defect in human cancer. Proc Natl Acad Sci USA.

[25] Abrams SI, Waight JD (2012). Identification of a G-CSF-Granulocytic MDSC axis that promotes tumor progression. Oncoimmunology..

[26] Salminen A, Kaarniranta K, Kauppinen A (2018). The role of myeloid-derived suppressor cells (MDSC) in the inflammaging process. Ageing Res Rev.

[27] Nam S, Kang K, Cha JS, Kim JW, Lee HG, Kim Y (2016). Interferon regulatory factor 4 (IRF4) controls myeloid-derived suppressor cell (MDSC) differentiation and function. J Leukoc Biol.

[28] Mackey JBG, Coffelt SB, Carlin LM (2019). Neutrophil maturity in cancer. Front Immunol.

[29] Keeley T, Costanzo-Garvey DL, Cook LM (2019). Unmasking the many faces of tumor-associated neutrophils and macrophages: considerations for targeting innate immune cells in cancer. Trends Cancer.

[30] Hawila E, Razon H, Wildbaum G, Blattner C, Sapir Y, Shaked Y (2017). CCR5 directs the mobilization of CD11b(+)Gr1(+)Ly6C(low) polymorphonuclear myeloid cells from the bone marrow to the blood to support tumor development. Cell Rep..

[31] Weichhart T, Saemann MD (2008). The PI3K/Akt/mTOR pathway in innate immune cells: emerging therapeutic applications. Ann Rheum Dis.

[32] Weichhart T, Costantino G, Poglitsch M, Rosner M, Zeyda M, Stuhlmeier KM (2008). The TSC-mTOR signaling pathway regulates the innate inflammatory response. Immunity..

[33] Jones LM, Broz ML, Ranger JJ, Ozcelik J, Ahn R, Zuo D (2016). STAT3 establishes an immunosuppressive microenvironment during the early stages of breast carcinogenesis to promote tumor growth and metastasis. Cancer Res.

[34] Vasquez-Dunddel D, Pan F, Zeng Q, Gorbounov M, Albesiano E, Fu J (2013). STAT3 regulates arginase-I in myeloid-derived suppressor cells from cancer patients. J Clin Invest.

[35] Cherfils-Vicini J, Iltis C, Cervera L, Pisano S, Croce O, Sadouni N (2019). Cancer cells induce immune escape via glycocalyx changes controlled by the telomeric protein TRF2. EMBO J.

[36] Schoggins JW, Wilson SJ, Panis M, Murphy MY, Jones CT, Bieniasz P (2011). A diverse range of gene products are effectors of the type I interferon antiviral response. Nature..

[37] McNab F, Mayer-Barber K, Sher A, Wack A, O’Garra A (2015). Type I interferons in infectious disease. Nat Rev Immunol.

[38] Liu YF, Zhuang KH, Chen B, Li PW, Zhou X, Jiang H (2018). Expansion and activation of monocytic-myeloid-derived suppressor cell via STAT3/arginase-I signaling in patients with ankylosing spondylitis. Arthritis Res Ther.

[39] Qi X, Jiang H, Liu P, Xie N, Fu R, Wang H (2021). Increased myeloid-derived suppressor cells in patients with myelodysplastic syndromes suppress CD8+ T lymphocyte function through the STAT3-ARG1 pathway. Leuk Lymphoma.

[40] Guchhait P, Tosi MF, Smith CW, Chakaraborty A (2003). The murine myeloid cell line 32Dcl3 as a model system for studying neutrophil functions. J Immunol Methods.

[41] Riffelmacher T, Clarke A, Richter FC, Stranks A, Pandey S, Danielli S (2017). Autophagy-dependent generation of free fatty acids is critical for normal neutrophil differentiation. Immunity..

[42] Palmer DC, Restifo NP (2009). Suppressors of cytokine signaling (SOCS) in T cell differentiation, maturation, and function. Trends Immunol.

[43] Elliott J, Johnston JA (2004). SOCS: role in inflammation, allergy and homeostasis. Trends Immunol.

[44] Lopez-Sanz L, Bernal S, Recio C, Lazaro I, Oguiza A, Melgar A (2018). SOCS1-targeted therapy ameliorates renal and vascular oxidative stress in diabetes via STAT1 and PI3K inhibition. Lab Invest.

[45] Sugase T, Takahashi T, Serada S, Fujimoto M, Ohkawara T, Hiramatsu K (2018). SOCS1 gene therapy has antitumor effects in imatinib-resistant gastrointestinal stromal tumor cells through FAK/PI3 K signaling. Gastric Cancer.

[46] Naka T, Narazaki M, Hirata M, Matsumoto T, Minamoto S, Aono A (1997). Structure and function of a new STAT-induced STAT inhibitor. Nature..

[47] Ortiz ML, Kumar V, Martner A, Mony S, Donthireddy L, Condamine T (2015). Immature myeloid cells directly contribute to skin tumor development by recruiting IL-17-producing CD4+ T cells. J Exp Med.

[48] Marvel D, Gabrilovich DI (2015). Myeloid-derived suppressor cells in the tumor microenvironment: expect the unexpected. J Clin Invest.

[49] Meyer C, Sevko A, Ramacher M, Bazhin AV, Falk CS, Osen W (2011). Chronic inflammation promotes myeloid-derived suppressor cell activation blocking antitumor immunity in transgenic mouse melanoma model. Proc Natl Acad Sci USA.

[50] Hsu BE, Tabaries S, Johnson RM, Andrzejewski S, Senecal J, Lehuede C (2019). Immature low-density neutrophils exhibit metabolic flexibility that facilitates breast cancer liver metastasis. Cell Rep.

[51] Kowanetz M, Wu X, Lee J, Tan M, Hagenbeek T, Qu X (2010). Granulocyte-colony stimulating factor promotes lung metastasis through mobilization of Ly6G+Ly6C+ granulocytes. Proc Natl Acad Sci USA.

[52] Waight JD, Hu Q, Miller A, Liu S, Abrams SI (2011). Tumor-derived G-CSF facilitates neoplastic growth through a granulocytic myeloid-derived suppressor cell-dependent mechanism. PLoS ONE.

[53] Amano MT, Castoldi A, Andrade-Oliveira V, Latancia MT, Terra FF, Correa-Costa M (2018). The lack of PI3Kgamma favors M1 macrophage polarization and does not prevent kidney diseases progression. Int Immunopharmacol.

[54] Kaneda MM, Messer KS, Ralainirina N, Li H, Leem CJ, Gorjestani S (2016). PI3Kgamma is a molecular switch that controls immune suppression. Nature..

[55] Borregaard N (2010). Neutrophils, from marrow to microbes. Immunity..

[56] Granot Z, Fridlender ZG (2015). Plasticity beyond cancer cells and the “immunosuppressive switch”. Cancer Res.

[57] Brandau S, Moses K, Lang S (2013). The kinship of neutrophils and granulocytic myeloid-derived suppressor cells in cancer: cousins, siblings or twins?. Semin Cancer Biol.

[58] Martinelli S, Urosevic M, Daryadel A, Oberholzer PA, Baumann C, Fey MF (2004). Induction of genes mediating interferon-dependent extracellular trap formation during neutrophil differentiation. J Biol Chem.

[59] Taleb KA-O, Auffray C, Villefroy P, Pereira AA-O, Hosmalin AA-O, Gaudry M (2017). Chronic type I IFN is sufficient to promote immunosuppression through accumulation of myeloid-derived suppressor cells. J Immunol.

[60] Ma H, Yang W, Zhang L, Liu S, Zhao M, Zhou G (2019). Interferon-alpha promotes immunosuppression through IFNAR1/STAT1 signalling in head and neck squamous cell carcinoma. Br J Cancer.

[61] Zhang CX, Ye SB, Ni JJ, Cai TT, Liu YN, Huang DJ (2019). STING signaling remodels the tumor microenvironment by antagonizing myeloid-derived suppressor cell expansion. Cell Death Differ.

[62] Zheng H, Qian J, Carbone CJ, Leu NA, Baker DP, Fuchs SY (2011). Vascular endothelial growth factor-induced elimination of the type 1 interferon receptor is required for efficient angiogenesis. Blood..

[63] Zheng H, Qian J, Varghese B, Baker DP, Fuchs S (2011). Ligand-stimulated downregulation of the alpha interferon receptor: role of protein kinase D2. Mol Cell Biol.

[64] Bhattacharya S, Qian J, Tzimas C, Baker DP, Koumenis C, Diehl JA (2011). Role of p38 protein kinase in the ligand-independent ubiquitination and down-regulation of the IFNAR1 chain of type I interferon receptor. J Biol Chem.

