# Supplementary material for: The downregulation of type I IFN signaling in G-MDSCs under tumor conditions promotes their development towards an immunosuppressive phenotype
Source: Cell Death Dis. 2022 Jan 10;13(1):36. doi: 10.1038/s41419-021-04487-w (PMC8748997; doi:10.1038/s41419-021-04487-w)
Supplement: Supplementary file 1 — Supplementary Figure legends [file 41419_2021_4487_MOESM1_ESM.docx]

**Supplementary Information**

**The downregulation of type I IFN signaling in G-MDSCs under tumor conditions promotes their development towards an immunosuppressive phenotype**

Yingying Sun, Xiaoqing Han, Chao Shang, Yawei Wang, Boya Xu, Shu Jiang, Yan Mo, Dake Wang, Yueshuang Ke*, Xianlu Zeng*.

**Content**

**Supplementary Figure legends**

Supplementary Fig. 1 The abnormally increased G-MDSCs under tumor conditions require further activation. (A) The absolute numbers of G-MDSCs in the peripheral blood of mice bearing tumors at different stages were counted. (B) The expression levels of the Arg1 and TGF-β genes in G-MDSCs in bone marrow taken from mice after different durations of tumor bearing were analyzed by semiquantitative Q-PCR. (C) The proliferation of T cells after coculture with bone marrow G-MDSCs taken from mice after different durations of tumor bearing was analyzed by flow cytometry. (D-E) The expression of CXCR2 and CD101 in G-MDSCs in bone marrow (BM) and peripheral blood taken from normal mice (Con-BL) and tumor-bearing mice (3W-BL) was analyzed by flow cytometry. Results are mean ± SD. Unpaired t test was used to determine significance (*p < 0.05, **p < 0.01, ***p < 0.001, and ****p < 0.0001).

**Supplementary Fig. 2** (A) Tumor-bearing mice were infused with either Con-G-MDSCs or 3Ws-G-MDSCs at the indicated time, and the absolute numbers of G-MDSCs in peripheral blood were counted. (B) The absolute numbers of G-MDSCs in peripheral blood were counted after anti-GR1 antibody treatment. Results are mean ± SD. Unpaired t test was used to determine significance (**p < 0.01 and ***p < 0.001).

Supplementary Fig. 3. (A) The purity of G-MDSC separated by magnetic beads was detected by flow cytometry. (B) The absolute numbers of G-MDSCs in peripheral blood were counted after injection of rapamycin (5 mg/kg) and stattic (4 mg/kg) into the tumor-bearing mice. Results are mean ± SD. Unpaired t test was used to determine significance (**p < 0.01).

Supplementary Fig. 4 The G-MDSCs in tumor tissue with the strongest immunosuppressive activity barely respond to IFN-I. (A) The expression of Arg1 in G-MDSCs in bone marrow (BM), peripheral blood (BL) and tumor tissue (tumor) taken from mice after 3 weeks of tumor bearing was analyzed by Q-PCR. (B-G) The expression of IFNAR and ISGs in G-MDSCs of different tissues (bone marrow, peripheral blood and tumor tissue) was analyzed by Q-PCR. (H-M) The expression of IFNAR and ISGs in G-MDSCs of different tissues (bone marrow, peripheral blood and tumor tissue) was analyzed by Q-PCR after incubation with IFNα for 2 h. Results are mean ± SD. Unpaired t test was used to determine significance (*p < 0.05 , **p < 0.01 and ***p < 0.001).

Supplementary Fig. 5 CXCL10 and CXCL13 did not directly affect the immunosuppressive function of G-MDSCs. (A) The expression levels of CXCL10, CXCL13 and G-CSF in B16-F10 cells were analyzed by Q-PCR. (B) The expression levels of CXCR3, CXCR5 and G-CSFR in G-MDSCs were analyzed by semiquantitative PCR. (C-D) The expression of Arg1 was analyzed by Q-PCR after incubation with different concentrations of CXCL10 and CXCL13 for 2 h. (E) The level of G-CSF in serum was analyzed by ELISA after different durations of tumor bearing. (F) The expression of G-CSF was analyzed by Q-PCR after mice were infected with lentiviruses carrying shScramble (shSCR), sh1-G-CSF or sh2-G-CSF (n=4). (G) The absolute numbers of G-MDSCs in peripheral blood were counted after mice were infected with lentiviruses carrying shSCR, sh1-G-CSF or sh2-G-CSF. Results are mean ± SD. Unpaired t test was used to determine significance (*p < 0.05, **p < 0.01, ***p < 0.001 and ****p < 0.0001).

Supplementary Fig. 6 G-CSF did not reduce the expression of ISGs in G-MDSCs. The expression of IFNAR (A-B) and ISGs (C-F) in G-MDSCs from the bone marrow of normal mice was analyzed by Q-PCR after incubation with different concentrations of G-CSF for 2 h. Results are mean ± SD. Unpaired t test was used to determine significance (*p < 0.05, **p < 0.01, and ***p < 0.001).

Supplementary Fig. 7 IFNAR interference in 32D cells inhibited the expression of ISGs. (A-D) The expression of ISGs in 32D clone 3 cells was analyzed by Q-PCR after incubation with IFNα (100 ng/ml) or 3W serum for 48 h, or after stimulation with IFNα for 24 h and subsequent incubation with 3W serum for 24 h (IFNα+3Ws-serum). (E-F) 32D clone 3 cells were transfected with siRNA targeting IFNAR or control for 36 h, and the interference efficiency was tested. (G-J) After IFNAR interference, the expression of ISGs in 32D clone 3 cells was analyzed by Q-PCR after incubation with IFNα (100 ng/ml). Results are mean ± SD. Unpaired t test was used to determine significance (*p < 0.05, **p < 0.01, ***p < 0.001 and ****p < 0.0001).

Supplementary Fig. 8 The IFN-I signal in mo-MDSCs was down regulated. (A) The expression of iNOS in mo-MDSCs of peripheral blood taken from mice bearing tumors for different durations was analyzed by Q-PCR. (B-G) The expression of IFNAR and ISGs in mo-MDSCs of peripheral blood taken from mice bearing tumors for different durations was analyzed by Q-PCR. Results are mean ± SD. Unpaired t test was used to determine significance (*p < 0.05, **p < 0.01, ***p < 0.001 and ****p < 0.0001).

**Supplementary Fig. 9** IFNα induced the expression of SOCS1. (A) The expression of SOCS1 in G-MDSCs of peripheral blood taken from mice after different durations of tumor bearing was analyzed by Q-PCR. (B-C) The expression of SOCS1 in G-MDSCs from the bone marrow of normal mice (B) and 32D clone 3 cells (C) was analyzed by Q-PCR after incubation with IFNα for 24 h. (D) The expression of SOCS1 in 32D clone 3 cells stimulated with IFNα was analyzed by Q-PCR after IFNAR interference. (E) 32D clone 3 cells were transfected with siRNA targeting SOCS1 or control for 36 h, and the interference efficiency was tested by Q-PCR. Results are mean ± SD. Unpaired t test was used to determine significance (*p < 0.05, **p < 0.01, ***p < 0.001 and ****p < 0.0001).
