# Supplementary figures and images for: The downregulation of type I IFN signaling in G-MDSCs under tumor conditions promotes their development towards an immunosuppressive phenotype
Source: Cell Death Dis. 2022 Jan 10;13(1):36. doi: 10.1038/s41419-021-04487-w (PMC8748997; doi:10.1038/s41419-021-04487-w)

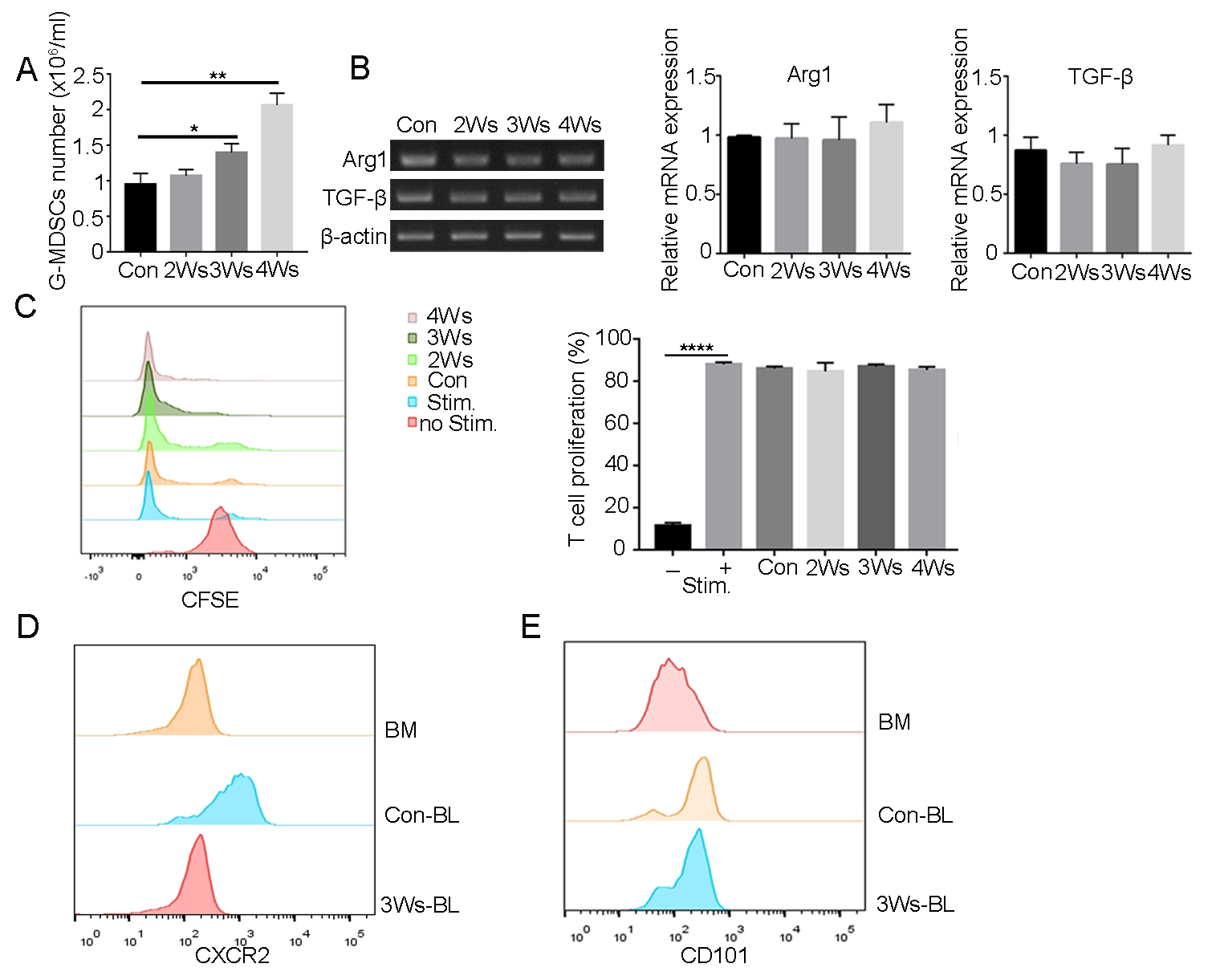

Supplement: Supplementary file 2 — Supplementary Fig. 1 [file 41419_2021_4487_MOESM2_ESM.tif]

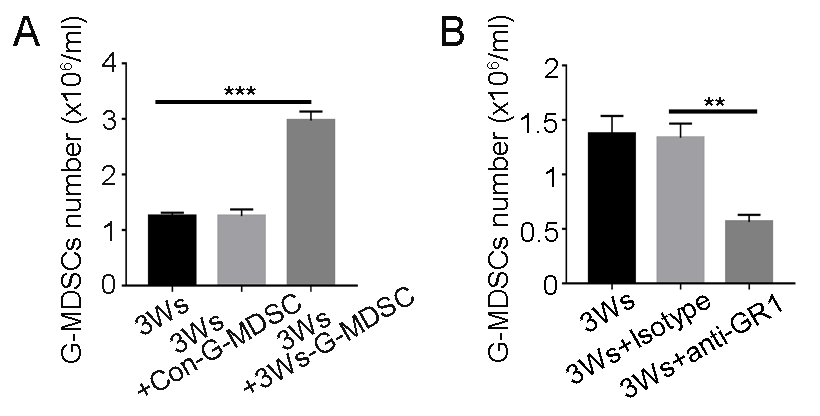

Supplement: Supplementary file 3 — Supplementary Fig. 2 [file 41419_2021_4487_MOESM3_ESM.tif]

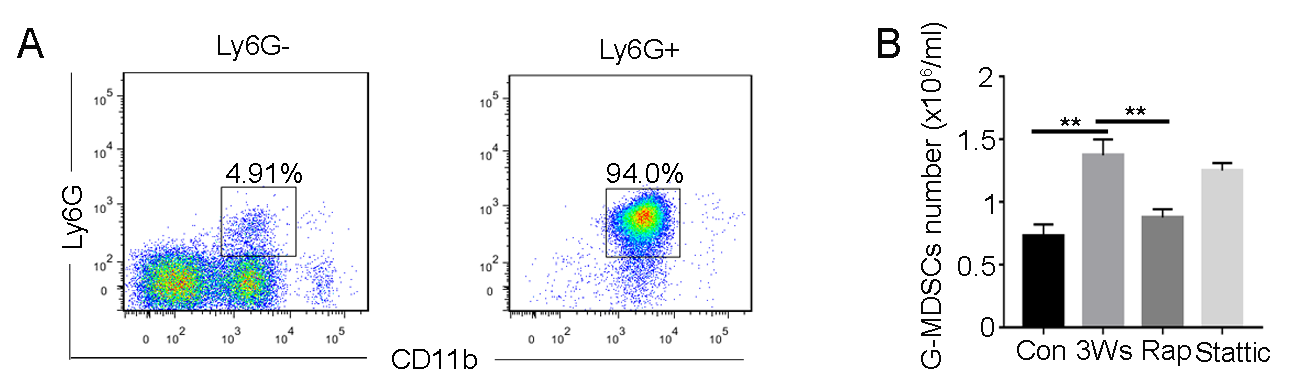

Supplement: Supplementary file 4 — Supplementary Fig. 3 [file 41419_2021_4487_MOESM4_ESM.tif]

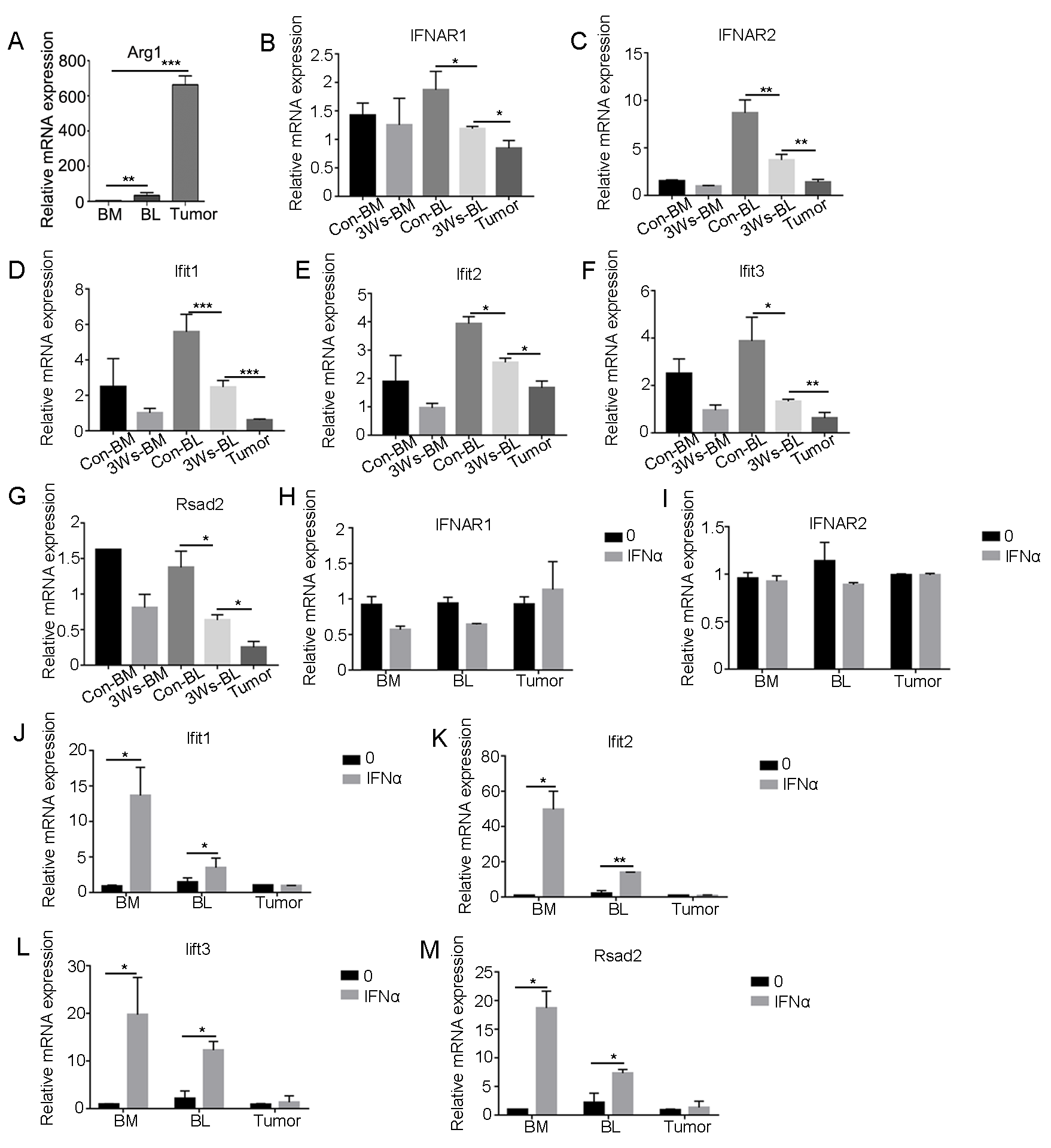

Supplement: Supplementary file 5 — Supplementary Fig. 4 [file 41419_2021_4487_MOESM5_ESM.tif]

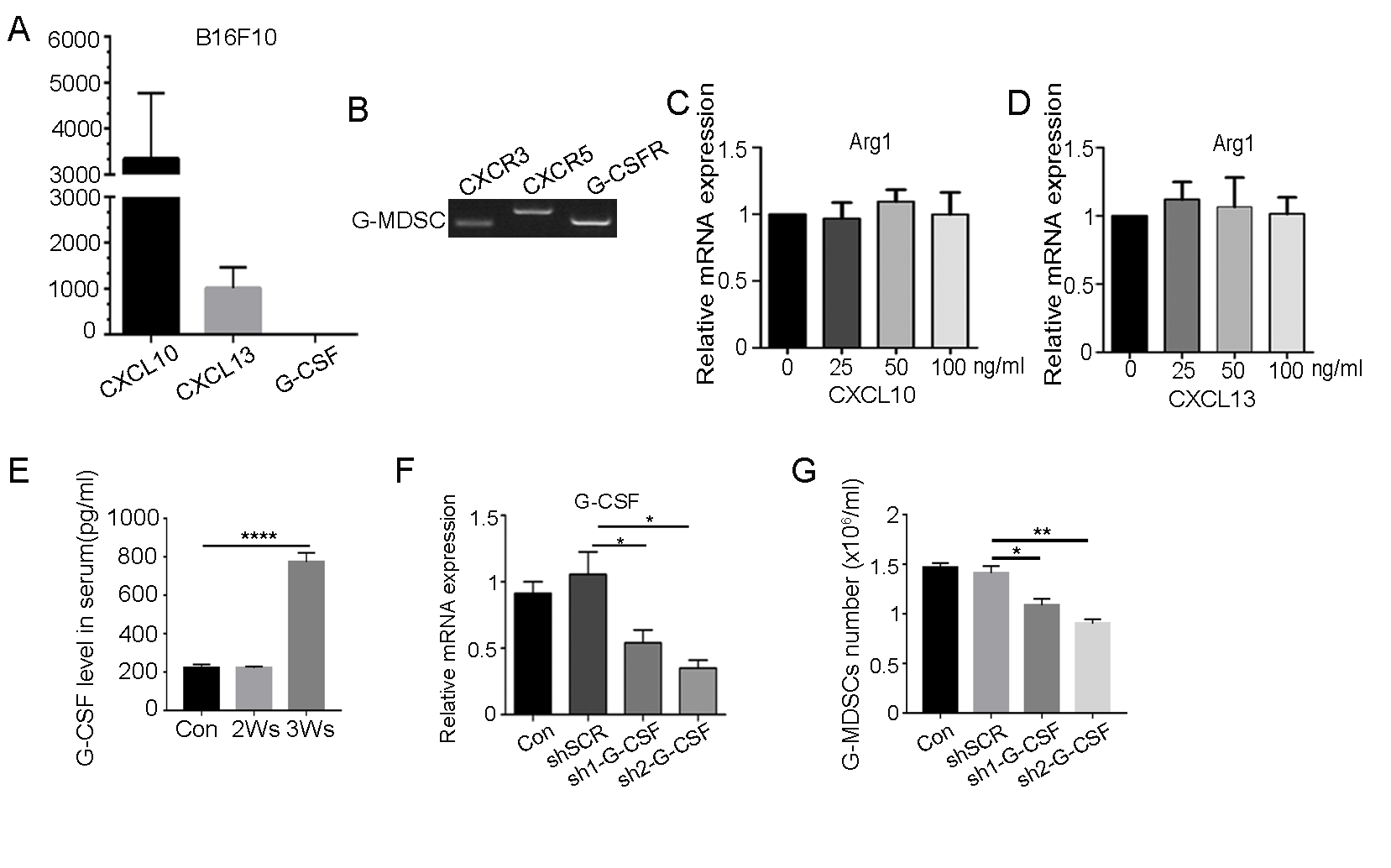

Supplement: Supplementary file 6 — Supplementary Fig. 5 [file 41419_2021_4487_MOESM6_ESM.tif]

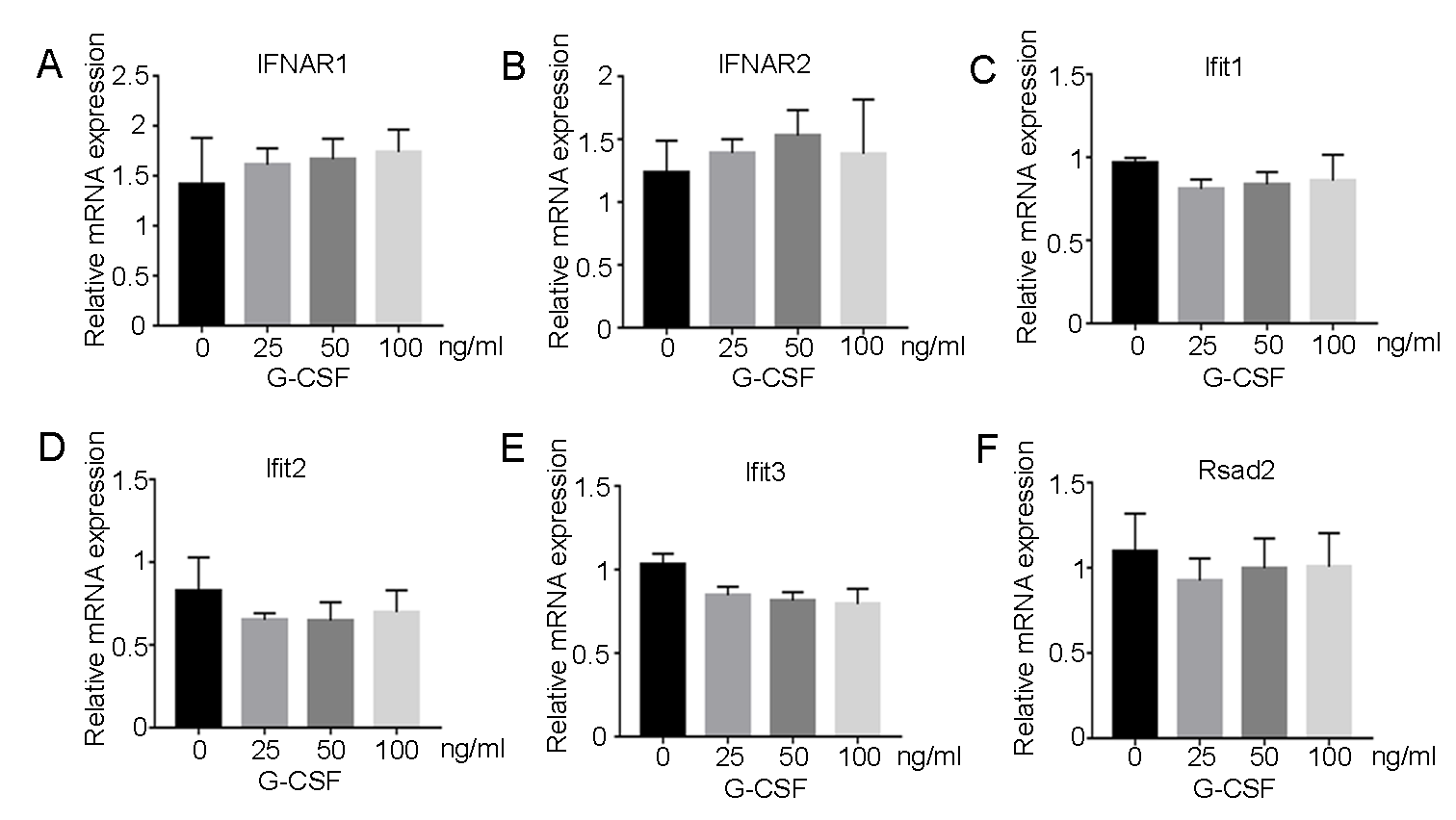

Supplement: Supplementary file 7 — Supplementary Fig. 6 [file 41419_2021_4487_MOESM7_ESM.tif]

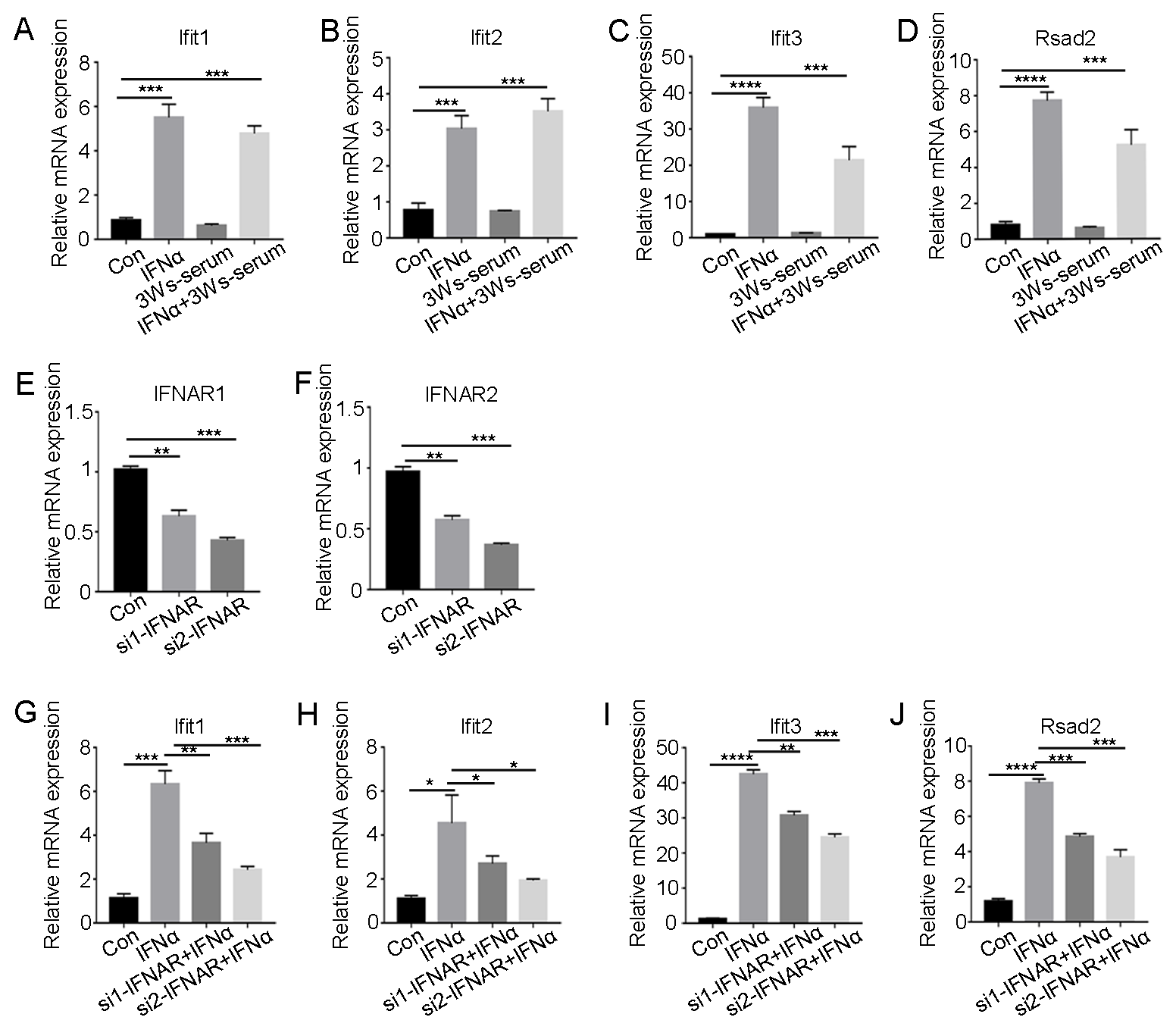

Supplement: Supplementary file 8 — Supplementary Fig. 7 [file 41419_2021_4487_MOESM8_ESM.tif]

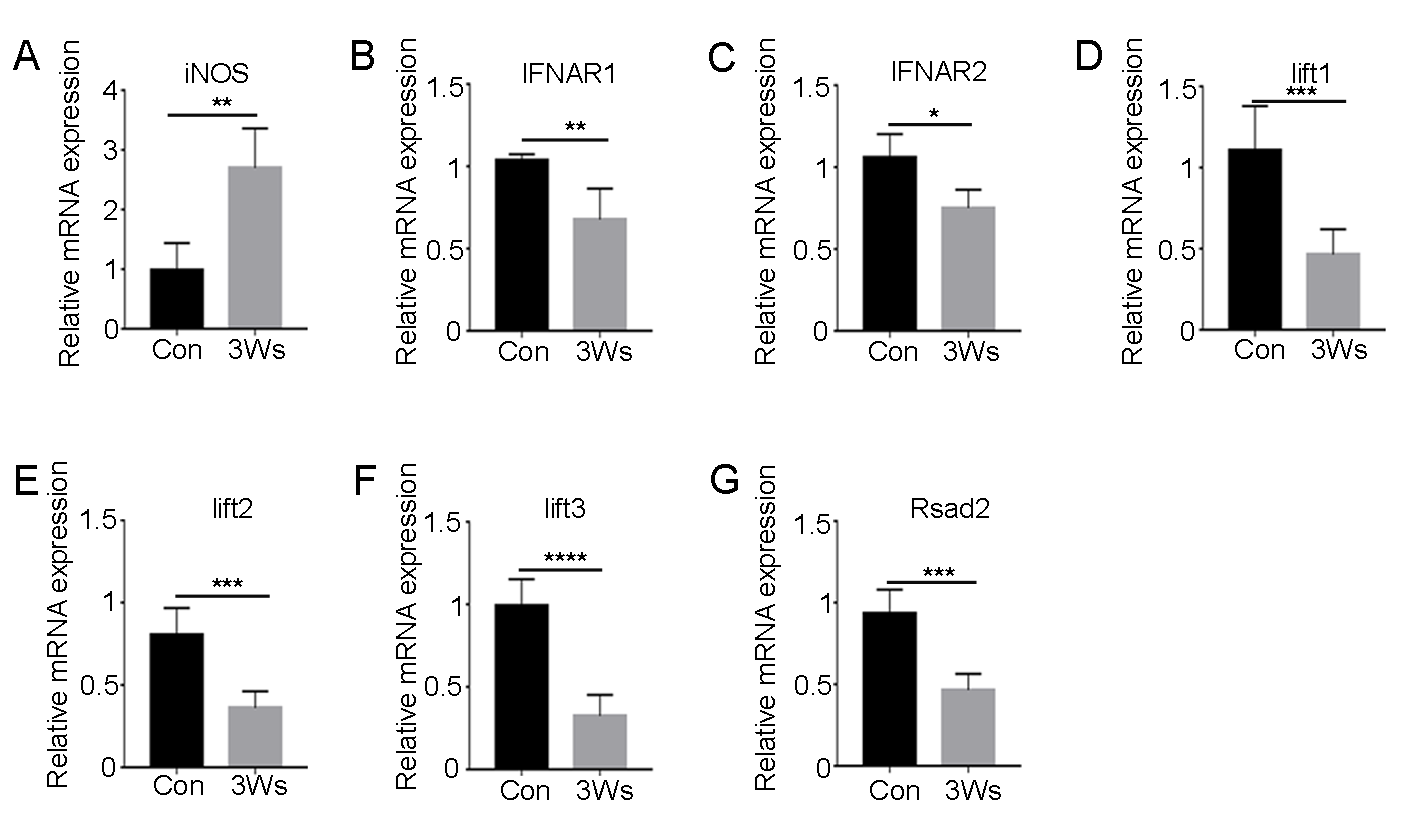

Supplement: Supplementary file 9 — Supplementary Fig. 8 [file 41419_2021_4487_MOESM9_ESM.tif]

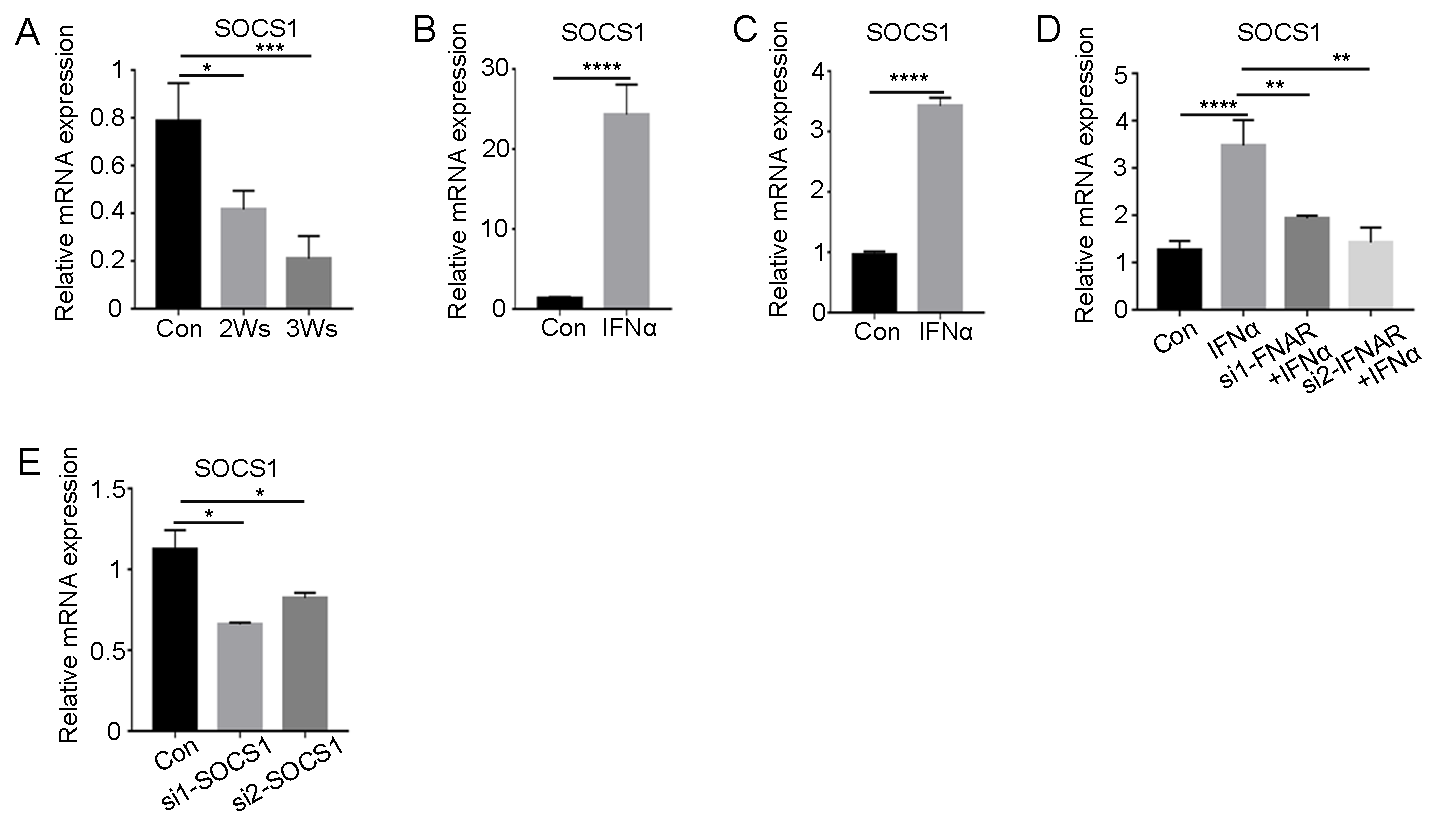

Supplement: Supplementary file 10 — Supplementary Fig. 9 [file 41419_2021_4487_MOESM10_ESM.tif]
